# Epigenomic analysis reveals prevalent contribution of transposable elements to *cis*-regulatory elements, tissue-specific expression, and alternative promoters in zebrafish

**DOI:** 10.1101/gr.276052.121

**Published:** 2022-07

**Authors:** Hyung Joo Lee, Yiran Hou, Ju Heon Maeng, Nakul M. Shah, Yujie Chen, Heather A. Lawson, Hongbo Yang, Feng Yue, Ting Wang

**Affiliations:** 1 Department of Genetics, Washington University School of Medicine, St. Louis, Missouri 63110, USA;; 2 Edison Family Center for Genome Sciences and Systems Biology, Washington University School of Medicine, St. Louis, Missouri 63110, USA;; 3 Department of Biochemistry and Molecular Genetics, Feinberg School of Medicine, Northwestern University, Chicago, Illinois 60611, USA;; 4 Robert H. Lurie Comprehensive Cancer Center of Northwestern University, Chicago, Illinois 60611, USA;; 5 McDonnell Genome Institute, Washington University School of Medicine, St. Louis, Missouri 63108, USA

## Abstract

Transposable elements (TEs) encode regulatory elements that impact gene expression in multiple species, yet a comprehensive analysis of zebrafish TEs in the context of gene regulation is lacking. Here, we systematically investigate the epigenomic and transcriptomic landscape of TEs across 11 adult zebrafish tissues using multidimensional sequencing data. We find that TEs contribute substantially to a diverse array of regulatory elements in the zebrafish genome and that 37% of TEs are positioned in active regulatory states in adult zebrafish tissues. We identify TE subfamilies enriched in highly specific regulatory elements among different tissues. We use transcript assembly to discover TE-derived transcriptional units expressed across tissues. Finally, we show that novel TE-derived promoters can initiate tissue-specific transcription of alternate gene isoforms. This work provides a comprehensive profile of TE activity across normal zebrafish tissues, shedding light on mechanisms underlying the regulation of gene expression in this widely used model organism.

Transposable elements (TEs) are highly repetitive DNA sequences comprising approximately half of mammalian genomes (International Human Genome Sequencing Consortium 2001; Mouse Genome Sequencing Consortium 2002). TEs can replicate themselves in host genomes, but the vast majority of mammalian TEs have lost their ability to transpose. Nevertheless, TE sequences can impact the regulation of host genetic material because they contain abundant transcription factor binding sites (Chuong et al. 2017; Sundaram and Wysocka 2020). To prevent potential damaging effects of TEs, various epigenetic mechanisms, including DNA methylation and repressive histone modifications, have evolved to suppress most TE activities (Slotkin and Martienssen 2007; Friedli and Trono 2015). Despite their prevalence and potential regulatory impact, the highly repetitive nature and low mappability of TEs have made them challenging to study with short-read sequencing techniques (Treangen and Salzberg 2012). Thus, TEs have often been ignored in genome-wide studies.

Mounting evidence has revealed that TEs serve as a rich source of functional regulatory elements in host genomes (Wang et al. 2007; Bourque et al. 2008; Feschotte 2008; Jacques et al. 2013; Sundaram et al. 2014; Ito et al. 2017; Pehrsson et al. 2019; Miao et al. 2020). TEs contribute to essential components of gene regulatory machinery in both humans and mice, including promoters, enhancers, and insulators. Specific TE subfamilies have rewired gene regulatory networks involved in many biological processes such as innate immune response (Chuong et al. 2016) and pregnancy (Lynch et al. 2011; Chuong et al. 2013). TEs can also act as tissue-specific enhancers (Xie et al. 2013; Todd et al. 2019) and chromatin boundaries (Schmidt et al. 2012; Choudhary et al. 2020). Further, TEs have been found to provide transcription start sites and exons to both protein-coding genes and noncoding RNAs, affecting variation in transcription in both normal and disease states (Kapusta et al. 2013; Thompson et al. 2016; Chishima et al. 2018; Jang et al. 2019; Pasquesi et al. 2020; Modzelewski et al. 2021).

Zebrafish is an important model organism for various research areas including development (Kimmel et al. 1995; Lee et al. 2015), human disease (Lieschke and Currie 2007; Kaufman et al. 2016), and regeneration (Gemberling et al. 2013; Lee et al. 2020). Zebrafish TEs have unique characteristics that are distinct from mammalian TEs (Supplemental Fig. S1). The most abundant TE class in zebrafish is the DNA transposon, comprising 34% of the genome (Howe et al. 2013). In human and mouse, DNA transposons occupy only 3% and 2% of the genome, respectively (International Human Genome Sequencing Consortium 2001; Mouse Genome Sequencing Consortium 2002). Only a handful of studies have investigated the roles of TEs in the zebrafish gene regulatory machinery. One example determined that EnSpm-N6, a fish-specific DNA transposon, can be a source of TP53 binding sites in the zebrafish genome (Micale et al. 2012). This is similar to the work revealing the contribution of a human-specific endogenous retrovirus to TP53 binding sites in the human genome (Wang et al. 2007). Additionally, it has been shown that zebrafish TEs can contribute to long noncoding RNAs (Kapusta et al. 2013). However, a systemic analysis of the contribution of zebrafish TEs to different classes of regulatory elements and the extent to which zebrafish TEs are expressed among tissues is lacking, leaving a substantial gap in our knowledge of zebrafish transcriptional regulation. Recently, zebrafish TE expression has been investigated in embryogenesis, shedding light on the pervasive TE transcription during development (Chang et al. 2022). Comprehensive profiling and analysis of TEs in the context of epigenetic states and gene regulatory networks can be achieved only with large epigenetic data sets across multiple tissues (Pehrsson et al. 2019). Recently, we generated the most comprehensive epigenomic profile of 11 adult zebrafish tissues and two embryonic tissues to date, including chromatin immunoprecipitation sequencing (ChIP-seq), transposase-accessible chromatin using sequencing (ATAC-seq), whole-genome bisulfite sequencing (WGBS), chromosome conformation capture (Hi-C), and RNA-seq (Yang et al. 2020). Together, these data empower us to investigate the *cis*-regulatory element (CRE) contribution of TEs in zebrafish.

Here, we intercalate these multidimensional transcriptome, epigenome, and three-dimensional genome structure data to create a comprehensive epigenetic and transcriptional landscape of zebrafish TEs across adult tissues. We characterize the TE landscape in zebrafish and their contribution to regulatory networks in both shared and tissue-specific fashions. Specifically, we explore TEs’ potential in forming regulatory elements and/or alternative transcripts and in interacting with surrounding genomic regions. We provide insights into the evolutionarily conserved phenomenon of TEs as a powerful source of regulatory function in host genomes.

## Results

### Epigenomic annotation of zebrafish transposable elements

**Figure 1.**
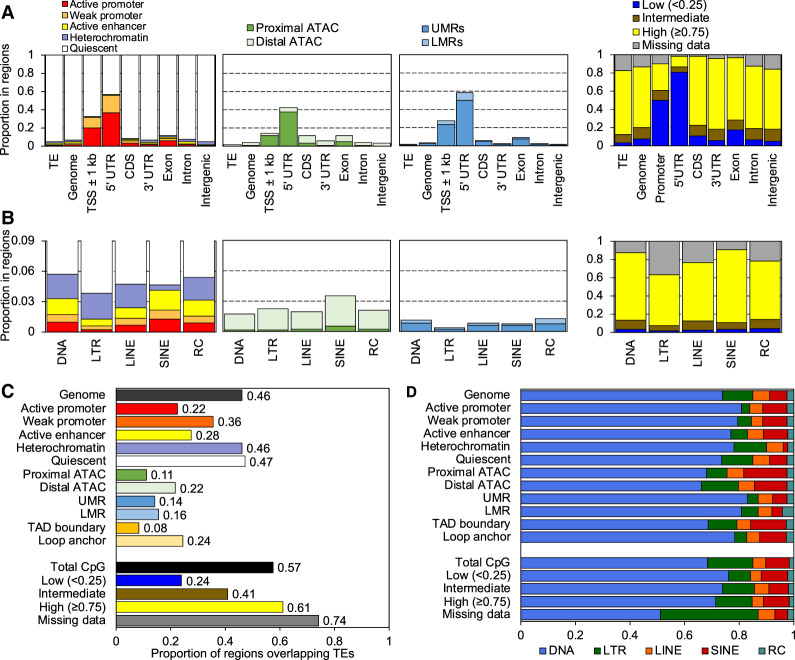
Substantial contribution of TEs to zebrafish CREs. (*A*) The proportion of bases within TEs, the entire genome, and Ensembl genic features annotated with each chromatin state (*leftmost*), ATAC-seq peak (*mid-left*), UMR or LMR (*mid-right*), and proportion of CpGs annotated with methylation state (*rightmost*), summed across all tissues with data for each category. (TSS) Transcription start site, (UTR) untranslated region, (CDS) coding sequences. (*B*) The proportion of bases within each TE class annotated by epigenetic state, summed across all tissues with data for each category. The color legend is the same as in *A*. (*C*) The total proportion of epigenetic states within TEs across all tissues versus the total proportion of all genomic bases and CpGs within TEs (black bars). (*D*) The proportion of each bar in *C* by TE class.

To profile the epigenetic landscape of TEs in zebrafish, we used the epigenetic states recently defined in 11 adult zebrafish tissues (Yang et al. 2020). These epigenetic states include five chromatin states (active and weak promoters, active enhancers, heterochromatin, and quiescent), proximal and distal ATAC-seq peaks, unmethylated and lowly methylated regions (UMRs and LMRs, respectively), and topologically associating domain (TAD) boundaries and loop anchors. Additionally, we used methylation levels to annotate CpGs in each tissue. We first compared the proportion of TEs in certain epigenetic states across all tissues to each TE's genomic proportions, as well as to proportions of genic features (Fig. 1A). As expected, transcription start sites (TSSs) and 5′ untranslated regions (UTRs) are enriched in active regulatory elements such as promoters, proximal ATAC-seq peaks, UMRs, and CpGs with low methylation levels. In contrast, TEs are depleted in these active regulatory states and enriched in highly methylated CpGs. For example, 0.90% of TE bases are annotated with an active promoter, whereas 37% of 5′ UTR bases are in the active promoter. A similar pattern was observed when the proportion of epigenetic states in TE and different genic feature bases were calculated using the union of epigenetic states across all tissues (Supplemental Fig. S2A). Whereas TEs are depleted in the active regulatory states, different classes of TEs show different epigenetic profiles (Fig. 1B; Supplemental Fig. S2B). For example, SINEs are positioned in more active enhancer states, distal ATAC-seq peaks, and TAD boundaries and loop anchors than other TE classes, whereas LTRs are positioned in more quiescent states and CpGs with missing methylation data. Overall, all TE classes are highly methylated, and only 1%–4% of CpGs in TEs are lowly methylated.

By comparing proportions from the opposite direction, we observed that TEs occupy a significant proportion of the zebrafish genome, encompassing 46% of total bases and 57% of CpGs (Fig. 1C). Further, TEs overlap a significant proportion of active regulatory regions. TEs comprise 22% of active promoters, 28% of active enhancers, 22% of distal ATAC-seq peaks, and 14% of UMRs (Fig. 1C). We also observed variation in the contribution of different TE classes (Fig. 1D). For example, SINEs comprise 16% of proximal and 12% of distal ATAC-seq peaks within TEs but only encompass 6.7% of TE bases. A similar pattern was observed in the union of epigenetic states across all tissues (Supplemental Fig. S2C,D). Taken together, these results show that TEs contribute substantially to active regulatory regions in zebrafish, despite being depleted in these regions. This implies that TEs play a vital role in shaping the regulatory machinery of the zebrafish genome, a result that is consistent with observations in mammals (Pehrsson et al. 2019).

### Dynamic epigenetic states of transposable elements across zebrafish tissues

**Figure 2.**
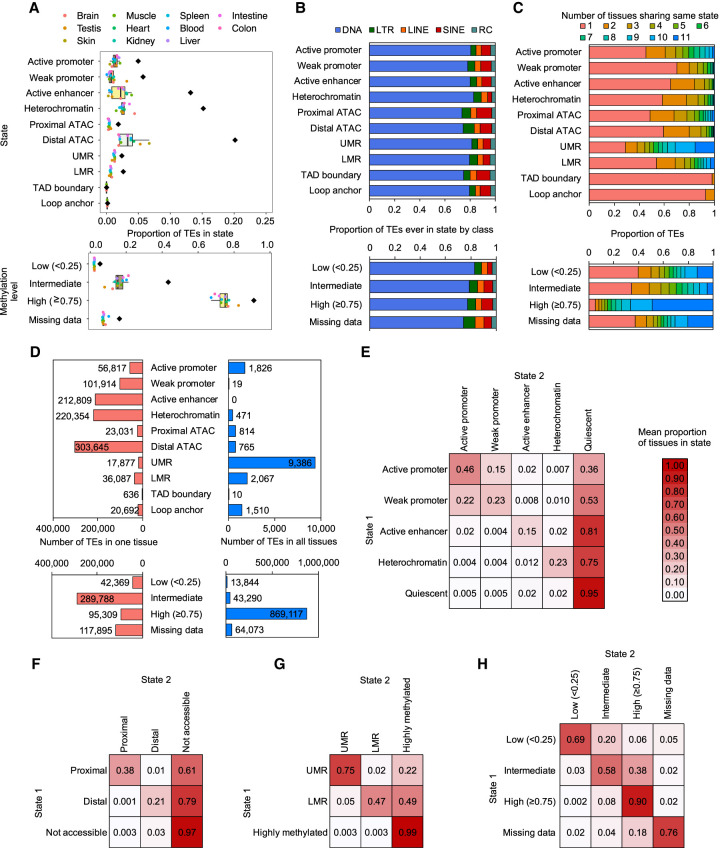
Epigenetic state dynamics of zebrafish TEs. (*A*) Boxplots indicate the proportion of all 2,532,468 individual TE fragments annotated by epigenetic state per tissue (*n* = 11 tissues, except for TAD boundary and loop anchor, *n* = 2). Black diamonds are the fraction of TEs annotated with the state in at least one tissue. For methylation level states, only TEs with CpGs are included (1,941,161 TE fragments, 77% of all TEs). (*B*) For TEs annotated with the epigenetic state in at least one tissue (*A*, black diamonds), the proportion in each TE class. (*C*) The proportion of TEs annotated with the same states across different tissues. (*D*) Number of TEs annotated by state only in one tissue (*left*) and annotated by state in all 11 tissues (in all, two tissues for TAD boundary and loop anchor). (*E*–*H*) For TEs in epigenetic State 1 in at least one tissue, the mean proportion of tissues in which they are annotated with epigenetic State 2 (represented by color scale). Different categories of epigenetic states, including chromatin states (*E*), ATAC-seq peaks (*F*), UMRs and LMRs (*G*), and methylation levels (*H*), are used.

To understand how the epigenetic states of TEs change across adult zebrafish tissues, we investigated the proportion of all 2,532,468 zebrafish TE fragments’ epigenetic annotation within each tissue. A small fraction (<10%) of individual TE fragments are positioned in active regulatory regions, including active promoters, weak promoters, active enhancers, ATAC-seq peaks, UMRs, and LMRs in a given tissue (Fig. 2A). However, a substantial fraction (37%) of TE fragments overlaps a potential regulatory region in at least one tissue. For example, a median of 2.4% of all individual TE fragments contribute to active enhancers, whereas 13% of TEs are in the active enhancer state in at least one tissue. Similarly, a median of 3.8% of TE fragments contribute to distal ATAC-seq peaks, whereas 20% of TEs are in distal ATAC-seq peaks in at least one tissue. To determine if the patterns we identified are due to chance, we shuffled genomic coordinates of TEs 20 times and investigated their overlay with epigenetic annotation across tissues (Methods). The fractions of shuffled TEs overlapping regulatory regions in at least one tissue are largely comparable with what we observed in true TEs (on average, 40.4% for any regulatory region, 13.5% for active enhancers, and 24.8% for distal ATAC-seq peaks). In contrast, ∼91% of TEs are highly methylated in at least one zebrafish tissue, which is higher than 68% estimated from shuffled TEs. These results are consistent with epigenetic annotations of human TEs using Roadmap Epigenomics Project data (Pehrsson et al. 2019). The majority of individual TEs contributing to various epigenetic states are DNA transposons, the primary class of zebrafish TE. However, there are certain classes overrepresented in specific epigenetic states. For example, although SINE elements comprise 7.0% of TE fragments in the zebrafish genome, they account for 12% of proximal ATAC-seq peaks and 11% of those in CCCTC-binding factors (CTCF) within TAD boundaries (Pearson's χ^2^ test, *P*-value < 2.2 × 10^−16^) (Fig. 2B). LTR elements (6.2% of all TEs) account for 8.7% of TEs in distal ATAC-seq peaks (Pearson's χ^2^ test, *P*-value < 2.2 × 10^−16^). These data suggest that different classes of TEs contribute to individual epigenetic states differently, with DNA transposons being the major contributor.

We next examined the dynamics of TEs’ epigenetic profiles across zebrafish tissues. Each TE is annotated with a specific epigenetic state across a number of different tissues. The majority of TEs annotated with active enhancers are found only in a single tissue, suggesting that active enhancer TEs are highly tissue-specific (Fig. 2C,D). Conversely, a substantial proportion of highly methylated TEs are annotated in all 11 adult tissues, indicating those TEs are universally methylated across tissues. Additionally, TEs within distal ATAC-seq peaks show more tissue specificity than those within proximal ATAC-seq peaks. TEs within LMRs show more tissue specificity than the ones with UMRs. This indicates that the observed tissue specificity of TEs’ epigenetic annotation reflects epigenetic state.

We further investigated the extent to which TEs are annotated with different epigenetic states over all tissues (Fig. 2E–H). For example, TEs in the active promoter state are in that state in 46% of tissues but are in the weak promoter state in 15% of tissues and are in the quiescent state in 36% of tissues (Fig. 2E). TEs in the active enhancer state are found in that state in 15% of tissues and are in the quiescent state in 81% of tissues. This suggests that TEs in promoter states in any tissue have a higher probability of also being in the promoter state in other tissues, whereas TEs in enhancer states are highly tissue-specific. Similarly, TEs in proximal ATAC-seq peaks and in UMR states are less tissue-specific than TEs in distal ATAC-seq peaks and in LMR states (Fig. 2F,G). Similar epigenetic state-dependent tissue specificity is seen in shuffled TEs (Supplemental Fig. S3). Taken together, these results indicate that TEs vary in epigenetic state across different tissues and that the tissue specificity of TE annotation varies across different epigenetic states.

Given the tissue specificity of TEs in enhancer states, we next asked to what extent TE enhancers in embryonic tissues remain active in adult tissues. We identified 17,202 TEs in enhancer regions of embryonic tissues and 52,541 TEs in enhancer regions of adult tissues (Supplemental Fig. S4). Among embryonic TE enhancers, 63.9% are also detected in adult tissues. This suggests that, in zebrafish, a large proportion of embryonic TE enhancers remain active and acquire tissue specificity in development. This result echoes recent findings in humans comparing hESC- and iPSC-derived neurons (Pontis et al. 2019).

### TE subfamily enrichment in active regulatory elements

**Figure 3.**
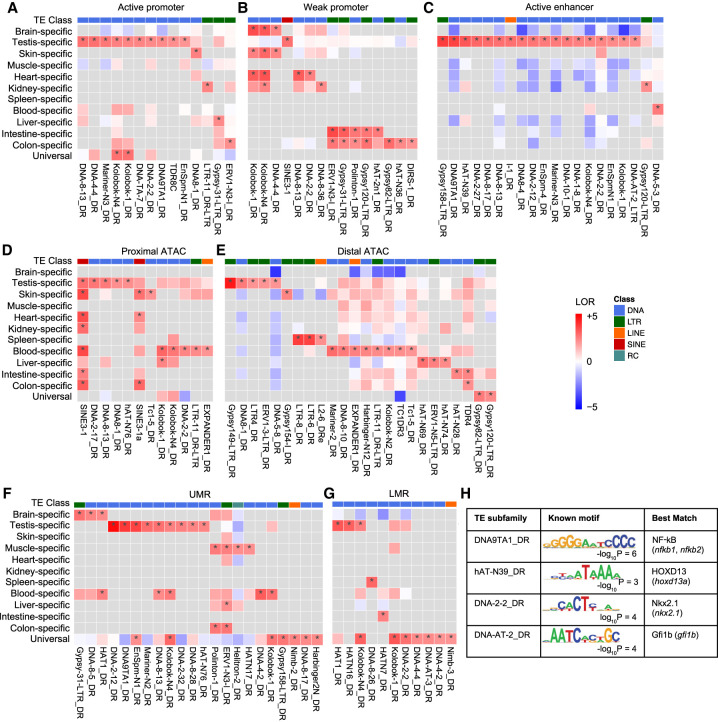
Heat map of tissue-specific enrichment of TE subfamilies in epigenetic states. (*A*) Active promoter. (*B*) Weak promoter. (*C*) Active enhancer. (*D*) Proximal ATAC-seq peaks. (*E*) Distal ATAC-seq peaks. (*F*) UMRs. (*G*) LMRs. (LOR) Log odds ratio, (*) FDR < 0.05 from permutation test with Benjamini–Hotchberg multiple testing corrections. (*H*) Known motifs of TE subfamilies enriched in testis-specific enhancer regions.

To further investigate tissue specificity, we investigated TE subfamily enrichment in active regulatory elements. To this end, we calculated the log odds ratio (LOR) enrichment of each TE subfamily in each tissue-specific or universal active regulatory element relative to the genomic background and found 152 enrichments with LOR > 2 (a fourfold enrichment over genomic background; Benjamini–Hochberg FDR < 0.05) (Fig. 3A–G). The enrichment pattern reveals TEs’ potential in contributing to the regulatory genome in both cross-tissue and tissue-specific ways. Four out of seven CRE categories (active enhancer, distal ATAC, UMRs, and LMRs) have at least one enriched TE subfamily in the universal elements, suggesting conserved regulatory roles of these TE subfamilies across tissues. In the proximal ATAC category, SINE3-1 and SINE3-1a are also enriched in multiple tissues (Fig. 3D), reflecting the significant contribution of SINEs to proximal ATAC-seq peaks as described above (Fig. 1D). In contrast, all seven categories have many TE subfamilies that are enriched in a tissue-specific manner relative to universal elements. For instance, 18, eight, and nine TE subfamilies are enriched in testis-specific active enhancer, blood-specific distal ATAC, and testis-specific UMRs (Fig. 3C,E,F), respectively, suggesting tissue-specific regulatory roles of these TEs.

Among all TEs overlapping active epigenetic states, the ones active in a testis-specific manner contribute to most of the subfamily level enrichment (Fig. 3A–G). Therefore, we focused on these TE subfamilies to determine if they are enriched for specific transcription factor binding motifs. We found that active TE elements from subfamilies DNA-AT-2_DR, DNA-2-2_DR, hAT-N39_DR, and DNA9TA1 show enrichment for Gfi1b, Nkx2.1, HOXD13, and NF-kB motifs, respectively (Fig. 3H; Supplemental Fig. S5A). Among the zebrafish transcription factors corresponding to these motifs, we found *nkx2.1b* expression is significantly higher in the testis sample compared to other adult tissues (Wilcoxon test, *P* < 0.05) (Supplemental Fig. S5B). Gene Ontology enrichment analysis with Nkx2.1 motif-containing TE fragments from DNA-2-2_DR subfamily suggests a functional association with response to hormone and estrogen (Supplemental Fig. S5C). Moreover, previous work in mice has shown that homologs of *nkx2.1b* and *hoxd13a* are associated with urogenital development, pointing to the possibility that these factors are involved in similar processes in zebrafish through TE-derived regulatory elements (Podlasek et al. 1997; Pakarinen et al. 2002).

We also analyzed TE subfamilies enriched for CTCF binding sites, which are critical for establishing 3D genome structure. CTCF binds chromatin at TAD boundaries and loop anchors (Rao et al. 2014; Tang et al. 2015). Therefore, we sought to identify TE subfamilies contributing to CTCF binding sites as putative genomic regions contributing to 3D genome architecture. We identified CTCF binding sites using footprint analysis with ATAC-seq data (Supplemental Fig. S6A,B) and found many TE subfamilies are enriched for CTCF-bound sites (Supplemental Fig. S6C). For example, SINE3-1a and HATN9_DR contributed 1016 and 647 CTCF-bound sites in total, respectively.

### Expression analysis of TE-derived transcripts

To investigate TE expression levels across different zebrafish tissues, we first used three classical approaches to allocate multimapped reads to genomic loci: equal distribution of multimapped reads, TEtranscripts (Jin et al. 2015), and SQuIRE (Yang et al. 2019). Due to TEs’ repetitive nature, many reads originating from TEs map to multiple locations of the genome. Multimapped reads are counted fractionally at genomic loci with the best alignments either by equal fraction or by expectation-maximization algorithms (TEtranscripts and SQuIRE). Overall, we found that TE expression profiles across tissues are consistent across the three methods and separate blood, embryonic tissue, and testis from the other tissues assayed (Supplemental Fig. S7A). We interrogated tissue-specific expression of TE subfamilies and identified 99, 96, and 103 TE subfamilies that show tissue-specific expression by equal fraction, TEtranscripts, and SQuIRE, respectively (Supplemental Fig. S7B). Among these, 37 TE subfamilies were consistently identified by all three methods. However, close examination of individual loci shows allocated reads spreading across intronic regions, reducing the confidence of representing the actual TE transcript structures (Supplemental Fig. S7C). This indicates that expression quantification through read allocation is limited at the subfamily level, resulting in high levels of noise at individual genomic sites.

**Figure 4.**
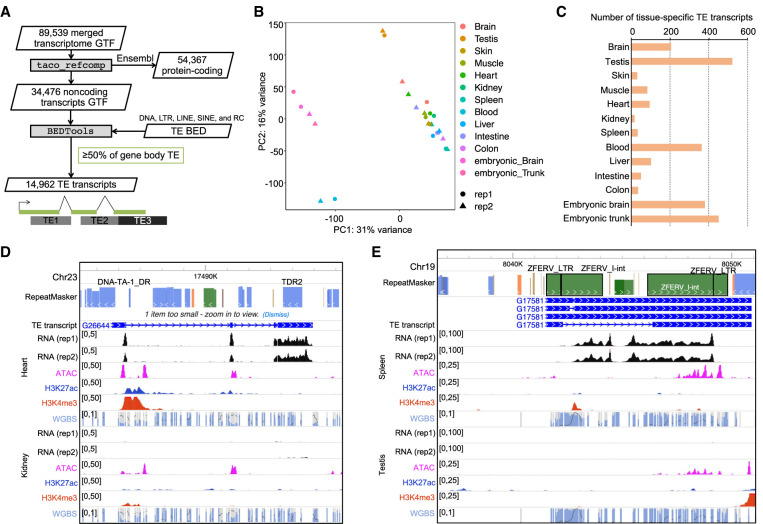
Expression of TE-derived transcripts. (*A*) Flowchart of methods used to identify TE-derived noncoding transcripts. (*B*) PCA plot of TE-derived transcripts. (*C*) Number of TE transcripts with tissue-specific expression. (*D*) Epigenome Browser view of TE transcript showing heart-specific expression. (*E*) Epigenome Browser view of TE transcript from the intact full-length *erv* (also known as *ZFERV*).

To address this issue, we used a transcript assembly approach that we recently developed (Modzelewski et al. 2021; Shao and Wang 2021). Briefly, we performed transcript assembly using all mapped RNA-seq reads and excluded all annotated protein-coding transcripts. We identified 14,962 noncoding transcripts that overlap TEs (Fig. 4A). The expression patterns of these TE transcripts again separate blood, embryonic tissues, and testis from the other tissues, which have more tissue-specific TE transcripts (Fig. 4B,C; Supplemental Fig. S8). We highlight two examples we identified: a novel noncoding transcript and an intact full-length endogenous retrovirus.

A novel noncoding transcript identified in Chromosome 23 shows heart-specific expression (Fig. 4D). The two overlapping DNA transposon elements, DNA-TA-1_DR and TDR2, fall in exons. This novel transcript originated from its own promoter, supported by the peak presence of ATAC-seq and ChIP-seq of H3K27ac and H3K4me3 and the absence of DNA methylation over the region in heart. An intact full-length endogenous retrovirus, *erv* (also known as *ZFERV*), encompasses the internal element and two LTRs in the spleen (Fig. 4E). *erv* has been previously reported as an intact full-length endogenous retrovirus expressed in the thymus (Shen and Steiner 2004). These results indicate that transcript assembly is a powerful approach to quantify TE expression levels. We show that zebrafish TEs contribute to tissue-specific expressed noncoding transcripts.

### Tissue-specific alternative promoters derived from TEs

**Figure 5.**
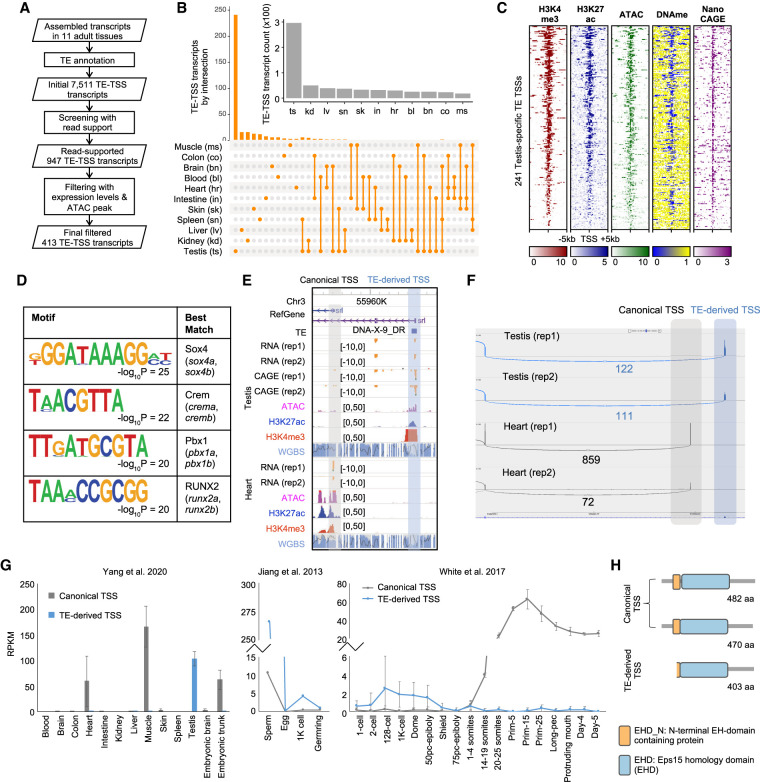
Tissue-specific alternative promoters derived from TEs. (*A*) Flowchart describing the methods used to identify TE-TSS transcripts. (*B*) UpSet plot of TE-TSS transcripts expressed in zebrafish tissue. (*C*) Heat maps of ChIP-seq, ATAC-seq signals, DNA methylation levels (DNAme), and nanoCAGE over 10-kb regions centered on testis-specific TE-derived TSSs. (*D*) Table of motifs enriched in testis-specific TE-derived alternative promoters, and the best TF matches. (*E*) Epigenome Browser view of TE-derived *srl* canonical and alternative promoters. (*F*) Sashimi plot showing RNA-seq reads spanning exon-exon junctions. Only reads anchored on the canonical exon 2 are shown for simplicity. (*G*) Plots of canonical and TE-derived TSS usages for *srl* in different tissues and developmental stages. (*H*) Protein structures from transcripts initiated from canonical TSS and putative protein structures from the TE-TSS transcript *srl*.

To investigate TEs’ contribution to tissue-specific alternative promoters, we identified novel TE-derived promoters. We used an approach similar to that previously used for the identification of TE onco-exaptation and TE-derived alternative promoters (Jang et al. 2019; Modzelewski et al. 2021). In brief, we used all assembled transcripts from the 11 adult tissues and identified 7511 transcripts whose 5′ ends are mapped to TEs (Fig. 5A). Next, we screened those transcripts by determining whether these promoters are supported by the RNA-seq reads. After filtering by expression level, we identified a total of 413 transcripts that originated from novel TE-derived TSSs (TE-TSS transcripts). The majority (328, 79%) of these transcripts are tissue-specific (Supplemental Fig. S9A). Testis comprises 73% of these tissue-specific transcripts followed by kidney and liver which each comprise 5% (Fig. 5B; Supplemental Fig. S9B). This testis specificity is likely due in part to epigenetic reprogramming occurring during gametogenesis (Reik et al. 2001).

We focused on the testis-specific TE-TSS transcripts. The TEs contributing to TSSs are mainly comprised of DNA classes (Supplemental Fig. S10A–C). To confirm TE promoter activity with an orthogonal approach, we generated nanoCAGE data from both testis and brain tissues. Of the 241 testis-specific TE-TSSs we identified, 85% are supported by reads covering the TSS, with 59% also supported by peaks (Supplemental Fig. S11A). The testis-specific TE-TSSs not supported by nanoCAGE peaks have lower RNA-seq expression levels compared to those with peaks, suggesting the lack of nanoCAGE peak is likely due to lower expression (Wilcoxon test, *P* < 0.05) (Supplemental Fig. S11B). Out of the six brain-specific TE-TSSs, three are supported by nanoCAGE data but there is not a significant correlation with RNA-seq expression level, likely due to small sample size (Supplemental Fig. S11C,D). We also examined epigenetic signatures indicative of active transcription. We found that testis-specific TE-TSSs have enriched signals of ATAC-seq, H3K4me3 and H3K27ac ChIP-seq signals only in the testis sample (Supplemental Fig. S12A–C). DNA demethylation signals are also observed in nontestis tissue samples (Supplemental Fig. S12D), suggesting testis-specific TE-TSSs may be primed in other tissues by DNA hypomethylation but not by chromatin regulation. Other tissue-specific TE-TSSs showed similar tissue-specific epigenetic landscape patterns (Supplemental Fig. S13A–D).

Next, to explore potential regulatory mechanisms of testis-specific TE-TSSs expression, we further investigated TF binding motifs enriched in these TE-TSSs. We performed known and de novo motif analyses and identified five enriched motifs with corresponding transcription factors expressed in testis samples (Methods; Fig. 5D; Supplemental Fig. S14A–E). Among these transcription factors, homologs of *sox4* and *pbx1* are known regulators of gonadal differentiation, suggesting TEs’ involvement in testis-specific gene regulation might be mediated by these transcription factors (Schnabel et al. 2003; Zhao et al. 2017).

One of the most highly expressed transcripts was a fusion transcript of DNA-X-9_DR and the gene encoding sarcalumenin (*srl*). In heart and muscle, the gene *srl* is transcribed from a previously annotated TSS that is not active in the testis. We found that in testis, *srl* is transcribed from the TE located in its upstream intergenic region, which contains a previously unannotated TSS (Fig. 5E). Using testis nanoCAGE peaks, we validated the promoter activity at this unannotated locus. The associated epigenetic signatures, including ATAC-seq, H3K27ac, and H3K4me3, further support this testis-specific promoter activity (Fig. 5E). The first exon originated from the TE-derived TSS and was spliced to a second exon and then again to the following exon, which is used as the second exon in the transcript, skipping the canonical first exon. The novel usage of this testis-specific promoter is further supported by the RNA-seq reads spanning the exon-exon junctions (Fig. 5F). We also investigated the promoter usage of the two TSSs by quantifying the number of RNA-seq reads mapped to the mutually exclusive exons. We found that the TE-derived TSS is used in a testis-specific manner (Fig. 5G). In addition, we used publicly available RNA-seq data to determine if the TE-derived TSS is used in the early developmental stages of the zebrafish embryos (Jiang et al. 2013; White et al. 2017). We found that the DNA-X-9_DR-derived TSS is used specifically in the sperm and in early embryos up to the shield stage (Fig. 5G). After the shield stage, usage of the canonical TSS for *srl* increases drastically, and usage of the TE-derived TSS is negligible. This result suggests that the TE-derived TSS is used in the early developmental stages of zebrafish embryos and the testis. Further, this TE-derived alternative promoter potentially produces an N-terminal truncated protein, implying that the testis-specific protein may function differently from the canonical *srl* protein (Fig. 5H). Whether or not this open reading frame can make stable protein in the testis and whether or not the N-terminal truncated protein has a unique function warrants future investigation.

The tissue-specific TE-derived alternative promoters are not limited to intergenic TEs. We observed many intronic TEs that also serve as tissue-specific alternative promoters. For example, the DNA transposon DNA-X-6_DR located in intron 11 of the *gpib* gene serves as a testis-specific alternative promoter and functions as a novel first exon (Supplemental Fig. S15A). The epigenetic signatures and the RNA-seq reads further support the promoter activity and the tissue-specific expression of the transcript (Supplemental Fig. S15A,B). Similarly, the DNA transposons hAT-N38_DR, DNA-2-20_DR, and DNA8-9_DR contribute to novel testis-specific usage of TSSs in the introns of the genes *ank3b*, *cyp2j20*, and *fez1*, respectively (Supplemental Figs. S16–S18).

Whereas most TE-derived alternative promoters were found in the testis and in DNA transposons, we also observed similar instances in other tissues and other classes of TE. For example, the DNA2-5_DR element serves as a brain-specific TSS for the gene *citb*, LTR-10_DR element serves as a kidney-specific TSS for the gene *add1*, and Polinton-1N1_DR serves as a blood-specific TSS for the gene *dnase1l4.1* (Supplemental Fig. S19A–I). Whereas these TSSs were not exclusively used over the canonical TSSs in the kidney or in the blood (Supplemental Fig. S19E,H), usage of these alternative promoters is supported by the epigenetic signatures and the RNA-seq reads.

Finally, we investigated TEs’ activities as a function of their evolutionary age. Sequence divergence-based age estimation shows that TE elements contributing to testis TE-TSSs are younger compared to other elements (Supplemental Fig. S20A), similar to observations made in human tissues (Pehrsson et al. 2019). However, little age difference was seen between TE elements overlapping active or inactive epigenetic states (Supplemental Fig. S20B), suggesting that the activity-age relationship is more complex than the current analysis resolution can detect.

## Discussion

We quantified substantial contribution of TEs to regulatory elements and the transcriptome of zebrafish across diverse tissues using comprehensive epigenomic and transcriptomic data encompassing 11 adult tissues and two embryonic tissues. In total, 37% of individual TE fragments in the zebrafish genome are annotated as active regulatory elements in at least one tissue. This analysis expands the roles of TEs in the evolution of gene regulation previously observed in mammalian genomes (Pehrsson et al. 2019) to the zebrafish genome. We found that various TE subfamilies belonging to different classes are enriched for different categories of tissue-specific active regulatory elements (Fig. 3). This suggests that TEs have been able to disseminate a battery of transcription factor binding sites throughout the genome, regardless of their transposition mechanisms, in line with the gene-battery model proposed by Britten and Davidson (Britten and Davidson 1969; Sundaram and Wang 2018).

Quantifying expression levels of TEs using second-generation sequencing data has been a challenge due to their repetitive nature (Lanciano and Cristofari 2020). Many RNA-seq reads originating from TEs are often discarded because they align to multiple genomic loci. Several computational tools have been developed to address this issue (Jin et al. 2015; Yang et al. 2019). These include assigning fractions of an ambiguously mapped read (multimapped reads) to each TE loci with an expectation-maximization algorithm and aggregating multimapped reads to the TE subfamily level. However, these approaches count RNA-seq reads at individual TEs or subfamilies and often fail to account for full-length transcript structure, where multiple TEs from different subfamilies can contribute. Here, we adapted the approach of using transcripts assembled from RNA-seq to quantify TE expression (Shao and Wang 2021). We show that this approach successfully identifies and quantifies novel noncoding RNA transcripts derived from multiple TE fragments of different subfamilies. Our approach further enables us to identify tissue-specific expression of TE-derived noncoding transcripts.

The transcript assembly approach also serves as an anchor for identifying TE-derived alternative promoters. Emerging evidence suggests that specific TEs can be exapted to provide promoter elements that reprogram host gene expression in various developmental and pathological processes (Feschotte 2008; Gardner et al. 2019; Tam et al. 2019; Miao et al. 2020). We recently showed the prevalence of TE onco-exaptation events across diverse cancer types (Jang et al. 2019). The best-characterized example is an intergenic TE *Alu*JB in human lung cancers that has been exapted to be an alternative promoter, up-regulating the oncogene *LIN28B*. The MIRb element located in the intronic region of the *ACE2* gene serves as an alternative promoter and generates a novel short ACE2 isoform in the airway epithelium, the main site of SARS-CoV-2 infection (Ng et al. 2020; Blume et al. 2021). In mammalian preimplantation embryos, species-specific TEs serve as alternative promoters to generate truncated Cdk2ap1 isoforms, suggesting that TE-derived alternative promoters can yield evolutionarily conserved alternative protein isoforms (Modzelewski et al. 2021). However, similar TE promoter usage in zebrafish had not been reported. In this study, we provided supports in both gene expression and nanoCAGE for the notion that TEs in the zebrafish genome can serve as alternative promoters and that the expression of those TE-derived isoforms can be highly tissue-specific.

The majority of the TE-derived alternative promoters in this study are testis-specific. The testis has one of the most complex, diverse, and rapidly evolving transcriptomes of all organs (Brawand et al. 2011; Soumillon et al. 2013). Regulation of gene expression in the germline is important to produce high-quality gametes, ensuring long-term maintenance of the species. However, what enables species-specific germline transcriptomes to evolve rapidly remains largely unexplored. Recently, a study exploring the mouse testis transcriptome discovered that endogenous retroviruses (ERVs) influence the germline transcriptome by contributing to many rapidly evolved active enhancers in mouse testis (Sakashita et al. 2020). In line with that, we provided supporting evidence of TEs as tissue-specific alternative promoters in zebrafish, most notably in testis.

We also show that these TE-derived testis-specific isoforms are present in sperm and early developmental stages. Fertilized zygotes are known to use maternal transcripts from the oocyte until the zygote genome is activated (Schulz and Harrison 2019). In zebrafish, zygote genome activation (ZGA) occurs 10 cell cycles after fertilization, and maternal transcripts are used until 3 h postfertilization. Our study suggests that cells in cycles prior to the ZGA possess not only maternal transcripts from oocyte but also paternal transcripts from sperm. This finding is in line with studies showing that paternal transcripts are transferred from sperm and exist in the early embryos (Boerke et al. 2007; Sendler et al. 2013; Hosken and Hodgson 2014). The specific biological and cellular functions that the novel TE-derived isoforms we discovered contribute to warrants further investigation. Many of the transcript isoforms we identified use TE-derived promoters exclusively. However, some genes have transcript isoforms from both TE-derived promoters and canonical promoters in the same tissue (Supplemental Fig. S15). The shared usages of canonical TSS and TE-derived TSS may be due to the different cell types present in tissues. A finer resolution of different cell types and single-cell analysis would further identify the specific cell types using TE-derived promoters.

In summary, our work represents an important synthesis of epigenomic and transcriptomic data in the context of TEs in the zebrafish genome. We showed that TEs contribute substantially to diverse tissue-specific regulatory elements and transcriptomes in zebrafish. Rapidly evolving technologies such as single-cell and genome/epigenome editing tools will further advance our knowledge on the biological function of TEs in zebrafish.

## Methods

### Zebrafish genome and epigenome data

All zebrafish epigenome data used in this study were previously generated (Yang et al. 2020). All analyses were performed using zebrafish genome assembly of GRCz10 (danRer10) and gene annotation Ensembl release 91 to be consistent with the functional annotation derived from the epigenome. We did not see much difference in the TE annotations between GRCz10 and the newer assembly GRCz11, so we did not realign the entire data set. Transposable elements used in this study were from five RepeatMasker-annotated repeats: DNA, LTR, LINE, SINE, and RC. For the epigenomic annotation of TEs, we used defined regulatory elements for each of 11 adult tissues. For the transcriptome analysis, we included two embryonic tissues. Raw RNA-seq sequencing data (NCBI Gene Expression Omnibus [GEO; https://www.ncbi.nlm.nih.gov/geo/] accession number GSE134055) were used for transcriptome analysis. ATAC-seq raw sequence (GEO; GSE134055) was used for the footprint analysis of CTCF.

### The intersection of TEs and epigenetic states

We used four different categories of epigenetic states previously defined (Yang et al. 2020). In brief, the four chromatin states (active promoters, weak promoters, active enhancers, and heterochromatin) were defined using histone ChIP-seq data following the order of active promoter (H3K27ac, H3K4me3, and ATAC-seq), weak promoter (H3K4me3 and ATAC-seq), active enhancer (distal H3K27ac and ATAC-seq), and heterochromatin (H3K9me2 or H3K9me3 sites). Genomic regions outside of these categories were assigned as quiescent states. Proximal and distal ATAC-seq peaks were defined using ATAC-seq data, where proximal peaks are regions overlapping regions 2.5 kb upstream of to 500 bp downstream from any transcription start site, and distal peaks are the remaining peaks. UMRs and LMRs were defined by WGBS data using no methylation and methylation <0.5 as our threshold, respectively (Burger et al. 2013). The CpGs were assigned as one of three states (low, intermediate, and high) according to methylation levels. The CpGs with a read coverage of less than five reads were considered missing data. In addition to the above four categories, we defined TAD boundary and loop anchor CTCF sites. TAD boundaries and loop anchors were previously defined in the different magnitudes of base-pair resolutions (40 kb and 25 kb, respectively) from the size of the TE fragments (Supplemental Fig. S1C), hampering the intersection analysis. To overcome this, we took advantage of the fact that TAD boundaries and loop anchors are enriched for the CTCF binding motifs. The TAD boundary CTCF sites were defined as the ATAC-seq peaks with CTCF motifs residing in the TAD boundaries. The loop anchor CTCF sites were defined as active regulatory elements (ATAC-seq peaks, active and weak promoters, and active enhancers) with CTCF motifs residing in loop anchors.

TEs, genome, and Ensembl genic features were intersected with each epigenetic state using BEDTools (Quinlan and Hall 2010), and overlapping base pairs were counted to calculate the proportion of bases in each state (Fig. 1A,B). Epigenetic states were intersected with TEs using BEDTools without regard to strand and were considered overlapping if they overlapped by ≥1 bp (Fig. 1C,D).

### Annotation of individual TE fragments with epigenetic states

All 2,532,468 individual TE fragments were annotated by epigenetic state per tissue. Each TE fragment was assigned to one state per each category. When a TE fragment overlaps more than one epigenetic state, the state with the highest number of base pairs sharing that TE fragment was chosen. To calculate DNA methylation levels of TE fragments, 1,941,161 TE fragments that have CpGs were used. A mean DNA methylation level of CpGs with a read coverage of five or more reads was calculated per TE fragment.

### TE shuffling

Genomic coordinates and class/family labels of all TEs were used as input for bedtools shuffle from BEDTools (Quinlan and Hall 2010) with default arguments for 20 iterations as conducted in a previous study (Pehrsson et al. 2019). Epigenetic state annotations and subsequent analyses were conducted in the same way as true TEs.

### TE subfamily enrichment analysis

TE subfamily enrichment was calculated as the log odds ratio as previously described (Sundaram et al. 2014; Pehrsson et al. 2019). Only subfamilies with >10 members in the CRE in the tissue were considered enriched (LOR > 2). Tissue-specific epigenetic states were defined as genomic regions in the corresponding state only in that tissue. Universal elements were defined as genomic regions that are in the corresponding epigenetic state in all adult tissues. For the CTCF-bound sites, footprint analysis was performed using CENTIPEDE (Pique-Regi et al. 2011) as previously described (Lee et al. 2020). The Tn5 insertion events from ATAC-seq in 200-bp windows around CTCF motif sites in the zebrafish genome were counted. These count matrices were then used as input for CENTIPEDE along with conservation scores (phastCons scores from eight-way vertebrate genome alignment, lifted over from Zv9 to GRCz10) at corresponding positions to predict the likelihood that each motif instance is bound by CTCF. The motif instances with a posterior probability greater than 0.95 were used as CTCF-bound sites. To confirm whether those sites were bound by CTCF, we used CTCF ChIP-seq data of zebrafish 24 h postfertilization (GEO; GSE133437) (Pérez-Rico et al. 2020). Heat maps of ATAC insert read counts and CTCF ChIP-seq signals over genome-wide CTCF motif sites were generated using deepTools (Ramírez et al. 2016). The ATAC-inferred CTCF-bound sites per tissue were used to calculate LOR for TE subfamily enrichment. To test the significance of TE subfamily enrichment in a specific tissue for certain epigenetic states, we binned the reference genome into 100-bp nonoverlapping windows using BEDTools (Quinlan and Hall 2010). We labeled each bin by their overlap with epigenetic state category. We conducted 1000 rounds of permutation to calculate statistical significance of enrichment. For each round of permutation, we shuffled category labels of bins and measured the overlap with TEs. We performed Benjamini–Hochberg FDR correction using TE subfamilies with >2 LOR.

### Motif enrichment analysis

For motif analysis focusing on TE fragments in testis-specific enhancer regions, we performed HOMER known motif analysis (Heinz et al. 2010) using their sequences as target regions, with TE fragments not located in enhancer regions as background. Motifs overlapping at least 10 TE fragments and having corresponding homolog transcription factors expressed in the testis sample were prioritized. Functional annotations over the same regions were conducted using Metascape with default settings (Zhou et al. 2019).

For motif analysis focusing on TEs contributing to testis-specific TE-TSSs, we performed both known and de novo motif analyses using HOMER (Heinz et al. 2010). We used 1-kb flanking windows of each TE-TSS as target regions. Subsequent criteria and analysis were performed as above.

### TE expression quantification benchmarking

To benchmark the TE expression quantification tools, we used the raw RNA-seq reads of 11 adult tissues and two embryonic tissues. First, adapter sequences were trimmed from the reads by using Trim Galore! (The Babraham Institute) version 0.6.1. The trimmed reads were directly used to run SQuIRE (Yang et al. 2019). For the equal fraction method and TEtranscripts, the trimmed reads were mapped to the zebrafish transcriptome (Ensembl release 91) and the zebrafish genome assembly (GRCz10) using STAR aligner (Dobin et al. 2013) version 2.7.2b with the following parameters: “‐‐outFilterMultimapNmax 500 ‐‐outFilterMatchNminOverLread 0.33 ‐‐outFilterScoreMinOverLread 0.33 ‐‐alignIntronMax 500000 ‐‐alignMatesGapMax 1000000 ‐‐alignSJDBoverhangMin 1 ‐‐sjdbOverhang 100”. By allowing outFilterMultimapNmax at 500, we can save almost all multimapped reads, rescuing them by allocation approaches or transcript assembly. BAM output files from the STAR aligner were used to run TEtranscripts (Jin et al. 2015). For the equal fraction method, the number of reads mapped to each TE fragment was summarized using featureCounts (Liao et al. 2014) version 2.0.0 with the following parameters: “-F GTF -t exon -g gene_id ‐‐extraAttributes transcript_id,family_id,class_id -O -M ‐‐fraction ‐‐primary -s 0 –p”. The TE fragment information was fed to featureCounts as a GTF format. Differential TE expression analysis was performed using DESeq2 (Love et al. 2014) version 1.18.1 with the resulting TE subfamilies × counts or TE fragments × counts matrices. TEs with fold change >2 and FDR < 0.05 were considered significantly differentially expressed from each pairwise comparison among 13 different tissues. Tissue-specific expression of TEs was assigned if a given TE showed more expression in a specific tissue in at least 10 pairwise comparisons.

### TE transcript assembly

Transcript assembly of each RNA-seq sample was performed as described previously. Briefly, StringTie2 (Kovaka et al. 2019) was used with the following parameters: “-j 2 -s 5 -f 0.05 -c 2”. To generate the master reference file, assembled transcripts from multiple RNA-seq samples were merged using TACO (Niknafs et al. 2017) with the default parameters. Protein-coding transcripts were excluded by comparing to Ensembl gene models using TACO's taco_refcomp command. Transcripts in which at least 50% of base pairs of exons overlap with TEs were defined as TE transcripts and used for expression quantification. The number of reads mapped to each TE transcript was summarized by using featureCounts (Liao et al. 2014) version 2.0.0 with the following parameters: “-F GTF -t exon -g transcript_id -O -M ‐‐fraction ‐‐primary”. Differential expression analysis of TE transcripts was performed using DESeq2 (Love et al. 2014) version 1.18.1 with the resulting transcripts × counts matrix. TE transcripts with fold change >2 and FDR < 0.05 were considered significantly differentially expressed from each pairwise comparison among 13 different tissues. Tissue-specific expression of TE transcripts was assigned if a TE transcript showed more expression in a specific tissue in at least 10 pairwise comparisons.

### Identification of TE-derived alternative promoters

We first assembled and annotated all the transcripts using the modified version of the TEProF pipeline for zebrafish study, similar to that used previously for human cancer data (Jang et al. 2019). In brief, the STAR-aligned BAM files were sorted and indexed. StringTie (Pertea et al. 2015) version 1.3.4d was used to assemble the BAM files for all the RNA-seq samples using the following parameters: ‐‐m 100 ‐‐c 1. These transcripts were then annotated with features from Ensembl release 91. The starting position of the transcript was annotated using RepeatMasker to find TE-derived TSSs. Then, the first exon of the transcript was annotated on the basis of overlap with exonic or intronic features from the Ensembl gene model. The assembled transcripts were aggregated across all the samples and the initial 5199 TE-TSS transcripts from 7173 instances were selected. These candidates were further filtered using read information, expression levels, and ATAC-seq signals. Only transcripts having at least 10 reads starting in the TE in the correct direction, at least one read going from the TE to the gene, and with the presence of ATAC peaks within 500-bp window from the 5′ end of the transcript were selected.

Heat maps of ChIP-seq and ATAC-seq signals along with DNA methylation levels over genomic regions around TE-derived TSSs were generated using deepTools (Ramírez et al. 2016). HOMER (Heinz et al. 2010) was used to perform motif enrichment analysis around TE-derived TSSs. Examples of TE-derived alternative promoters were visualized using the WashU Epigenome Browser (Li et al. 2019). The Integrative Genomics Viewer (Robinson et al. 2017) was used to draw Sashimi plots (Katz et al. 2015) visualizing RNA-seq reads spanning splice junctions. To quantify the number of RNA-seq reads mapped to TE-derived alternative promoters in the various developmental stages of the zebrafish embryos, we downloaded raw RNA-seq reads from GEO accession number GSE44075 (Jiang et al. 2013) and the NCBI BioProject database (https://www.ncbi.nlm.nih.gov/bioproject/) accession number PRJEB12982 (White et al. 2017). Reads mapping to the canonical exons or exons with the TE-derived promoters were extracted and used to calculate RPKM values.

### Adult zebrafish tissue nanoCAGE-seq

Tübingen zebrafish at 6 mo of age were euthanized in icy water and dissected to separate testis or brain tissues. All tissues collected were washed in 1× PBS, then flash-frozen on dry ice. Tissue chunks from one fish were considered as one replicate. For RNA extraction, we lysed tissues in TRIzol reagent and separated out the aqueous phase following the manufacturer's protocol (Invitrogen). RNA was extracted from the aqueous phase using RNA Clean & Concentrator-5 (Zymo). Poly(A)^+^ RNAs were isolated from total RNA using a Dynabeads mRNA Purification kit. NanoCAGE library preparation was performed as previously described using 50 ng of poly(A)^+^ RNAs for each replicate (Poulain et al. 2017).

### Transposable element age estimations

To estimate zebrafish TE age, we obtained alignment and output files of GRCz10 genome from RepeatMasker-4.0.6 and calculated Jukes–Cantor distance from substitutions in the alignments between each TE and its consensus sequence as described previously (Choudhary et al. 2020). TEs with a substitution rate >50% were excluded from downstream analysis due to high probability of misalignment.

### NanoCAGE analysis

Raw nanoCAGE sequencing data were processed as follows: Template switching oligos and UMIs were trimmed by Tagdust (version 2.33) with parameters “-1 O:N -2 F:NNNNNNNNN -3 S:TATAGGG -4 R:N -d 10000 –show_finger_seq”. Trimmed reads were aligned to the reference genome (GRCz10) using STAR (version 2.5.4b) with parameters “‐‐runMode alignReads ‐‐twopassMode Basic ‐‐chimOutType WithinBAM SoftClip” and GENCODE gene annotation (version 91). Only uniquely mapped and deduplicated reads were retained and converted into CTSS files using an in-house script. CTSS files were run through CAGEr (version 1.28) for peak calling as previously described (Brocks et al. 2017). We adjusted one parameter (nrPassThreshold = 1 for “clusterCTSS” function) from Brocks’ workflow. To eliminate spurious peaks, an in-house script implementing CapFilter was used with the minimum cutoff of 0.3 (Cumbie et al. 2015).

### NanoCAGE support of tissue-specific TE-TSSs

To find tissue-specific TE-TSSs supported by nanoCAGE data, the closest distance from tissue-specific TE-TSSs and the nanoCAGE signal was calculated using “bedtools closest” (parameters: “-s -d -t first -a < tissue specific TE-TSSs > -b”). A tolerance window of 100 bp was used to determine TE-TSSs supported by nanoCAGE peaks. The same analysis was repeated at the level of reads using unencoded G reads.

## Data access

All raw data generated in this study have been submitted to the NCBI BioProject database (https://www.ncbi.nlm.nih.gov/bioproject/) under accession number PRJNA799647. All data generated in this study can be visualized in the WashU Epigenome Browser (https://epigenome.wustl.edu/zebrafishENCODE/). All custom scripts used to perform the analysis in this study are available at GitHub (http://github.com/twlab/zebrafish_TE_epignome) and as Supplemental Code.

## Supplementary Material

Supplemental Material
