# Supplementary material for: Epigenomic analysis reveals prevalent contribution of transposable elements to cis-regulatory elements, tissue-specific expression, and alternative promoters in zebrafish
Source: Genome Res. 2022 Jul;32(7):1424–36. doi: 10.1101/gr.276052.121 (PMC9341505; doi:10.1101/gr.276052.121)
Supplement: Supplemental Material [file supp_32_7_1424__DC1.html]

Epigenomic analysis reveals prevalent contribution of transposable elements to cis-regulatory elements, tissue-specific expression, and alternative promoters in zebrafish — Epigenomic analysis reveals prevalent contribution of transposable elements to cis-regulatory elements, tissue-specific expression, and alternative promoters in zebrafish — Supplemental Material 

# Epigenomic analysis reveals prevalent contribution of transposable elements to *cis*-regulatory elements, tissue-specific expression, and alternative promoters in zebrafish

## Supplemental Material

- Supplemental\_code.zip
- Supplemental\_Material.pdf
