# Supplementary material for: Epigenomic analysis reveals prevalent contribution of transposable elements to cis-regulatory elements, tissue-specific expression, and alternative promoters in zebrafish
Source: Genome Res. 2022 Jul;32(7):1424–36. doi: 10.1101/gr.276052.121 (PMC9341505; doi:10.1101/gr.276052.121)
Supplement: Supplemental Material [file supp_gr.276052.121_Supplemental_Material.pdf]

SUPPLEMENTAL MATERIALS

Epigenomic analysis reveals prevalent contribution of transposable elements to *cis*-regulatory elements, tissue-specific expression, and alternative promoters in zebrafish

Hyung Joo Lee, Yiran Hou, Ju Heon Maeng, Nakul M. Shah, Yujie Chen, Heather A. Lawson, Hongbo Yang, Feng Yue, and Ting Wang

Table of Contents

Supplemental Figure S1. Characteristics of the zebrafish TEs.

Supplemental Figure S2. Substantial contribution of TEs to the zebrafish CREs.

Supplemental Figure S3. Dynamics epigenetic state transitions of shuffled TE.

Supplemental Figure S4. Common and unique TE-derived enhancers between adult and embryonic tissues.

Supplemental Figure S5. Motif analysis of enriched TE subfamilies in testis-specific enhancer regions.

Supplemental Figure S6. TE subfamily enrichment for the CTCF binding sites.

Supplemental Figure S7. TE subfamily-level expression quantification benchmarking.

Supplemental Figure S8. Tissue-specific expression level of TE transcripts.

Supplemental Figure S9. Number of tissues expressing TE transcripts.

Supplemental Figure S10. Testis-specific alternative promoters derived from TEs.

Supplemental Figure S11. nanoCAGE validation of testis-specific and brain-specific TE-TSS.

Supplemental Figure S12. Epigenetic landscape of testis-specific TE-TSS across all tissues.

Supplemental Figure S13. Epigenetic landscape of non-testis tissue-specific TE-TSS across all tissues.

Supplemental Figure S14. Motif analysis of testis-specific TE-TSSs.

Supplemental Figure S15. Testis-specific TE-TSS transcript of *gpib*.

Supplemental Figure S16. Testis-specific TE-TSS transcript of *ank3b*.

Supplemental Figure S17. Testis-specific TE-TSS transcript of *cyp2j20*.

Supplemental Figure S18. Testis-specific TE-TSS transcript of *fez1*.

Supplemental Figure S19. Tissue-specific TE-TSSs transcripts in non-testis samples.

Supplemental Figure S20. Age distribution of TEs contributing to TE-TSS and CREs.

Supplemental Figure S1

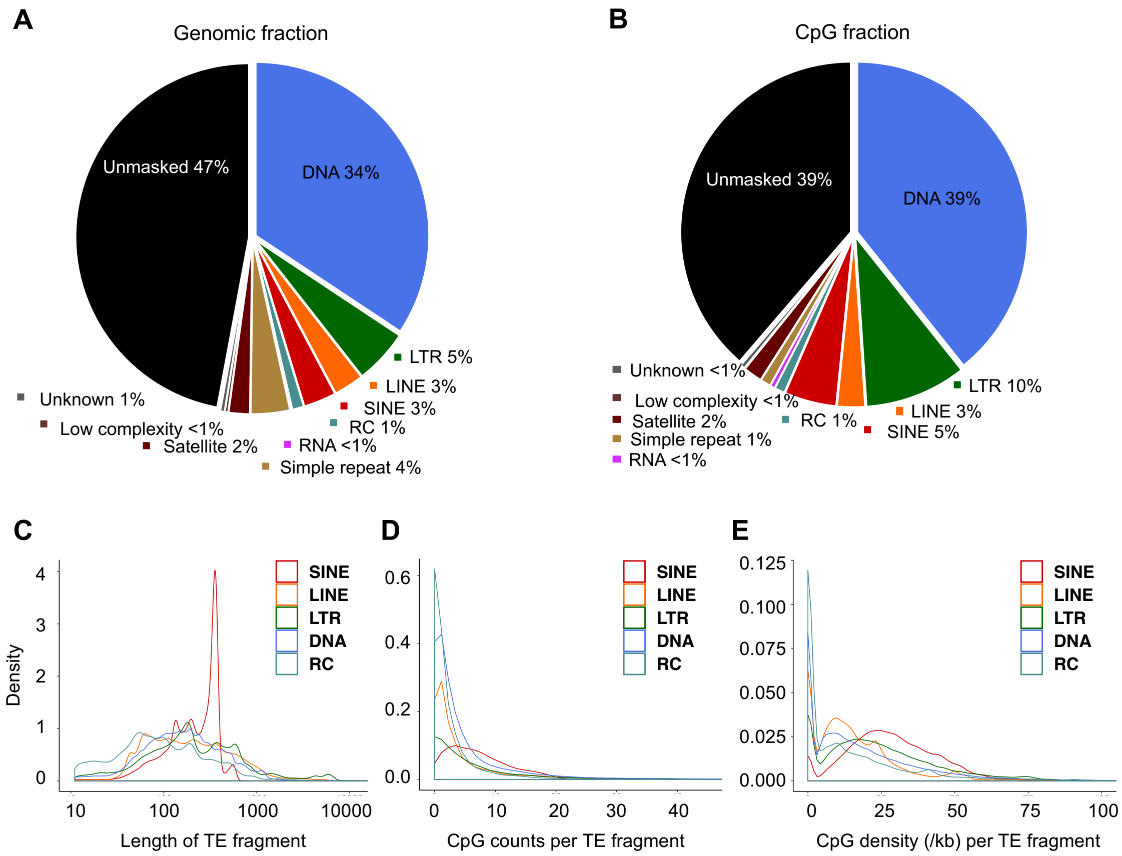

Supplemental Figure S1. Characteristics of the zebrafish TEs. (*A*) The fraction of the zebrafish genome by the class of all repetitive elements. The number of base pairs each class occupies in the zebrafish genome was used to calculate the fractions. (*B*) The CpG fractions in the zebrafish genomes by the class of all repetitive elements. The number of CpGs located in each class was used to calculate the fractions. (*C*) The distribution plot of the length of TE fragments by class in the zebrafish genome. (*D*) The distribution plot of the CpG counts in TE fragments by class in the zebrafish genome. (*E*) The distribution plot of the CpG density of TE fragments by class in the zebrafish genome.

Supplemental Figure S2

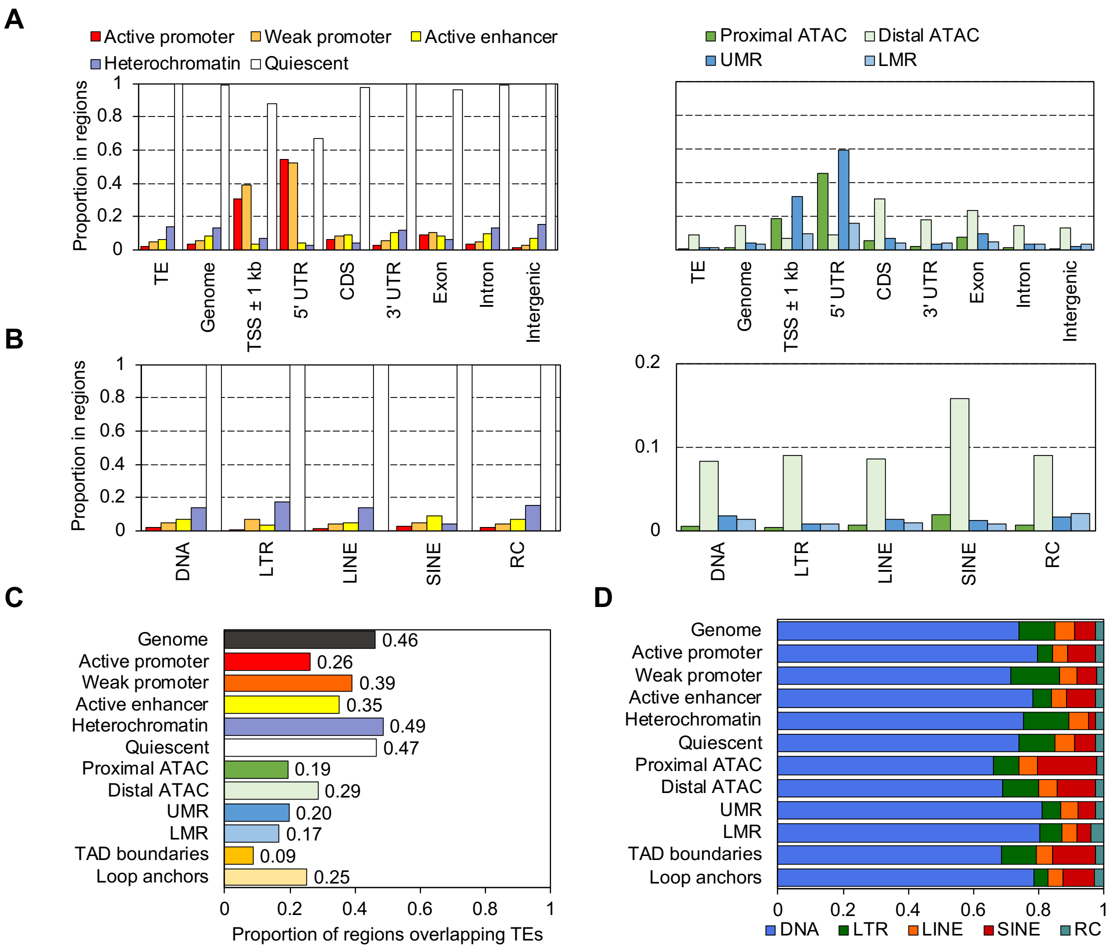

Supplemental Figure S2. Substantial contribution of TEs to zebrafish CREs. (*A*) The proportion of bases within TEs, the entire genome, and Ensembl genic features annotated with each chromatin state (leftmost), ATAC-seq peak (mid-left), UMR or LMR (mid-right), and proportion of CpGs annotated with methylation state (rightmost), using the union regions across all tissues. TSS: transcription start site; UTR: untranslated region; CDS: coding sequences. (*B*) The proportion of bases within each TE class annotated by epigenetic state, using the union regions across all tissues. The color legend is the same as (*A*). (*C*) The total proportion of the epigenetic states, union across all tissues, within TEs across all tissues vs. the total proportion of all genomic bases and CpGs within TEs (black bars). (*D*) The proportion of each bar in (*C*) by TE class.

Supplemental Figure S3

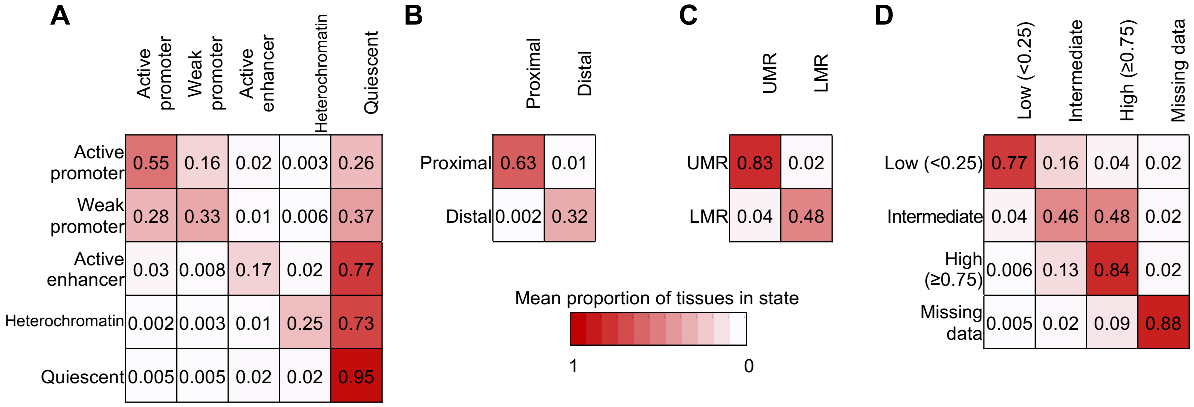

Supplemental Figure S3. Dynamics epigenetic state transitions of shuffled TE. For shuffled TEs in epigenetic State 1 in at least one tissue, the mean proportion of tissues in which they are annotated with epigenetic State 2 (represented by color scale). Different categories of epigenetic states, including chromatin states (*A*), ATAC-seq peaks (*B*), UMRs and LMRs (*C*), and methylation levels (*D*), are used.

Supplemental Figure S4

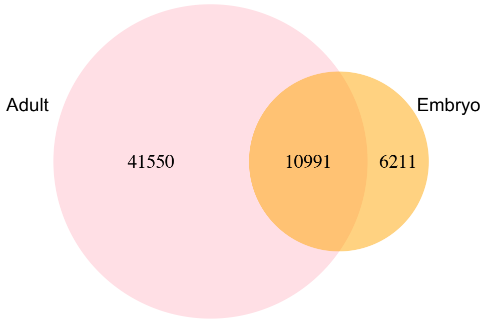

Supplemental Figure S4. Common and unique TE-derived enhancers between adult and embryonic tissues. Venn diagram showing the number of TE-derived enhancers that are unique or shared between adult and embryonic tissues. All adult by-tissue identified enhancers are pooled into a union set for adult enhancers, as are embryonic enhancers.

Supplemental Figure S5

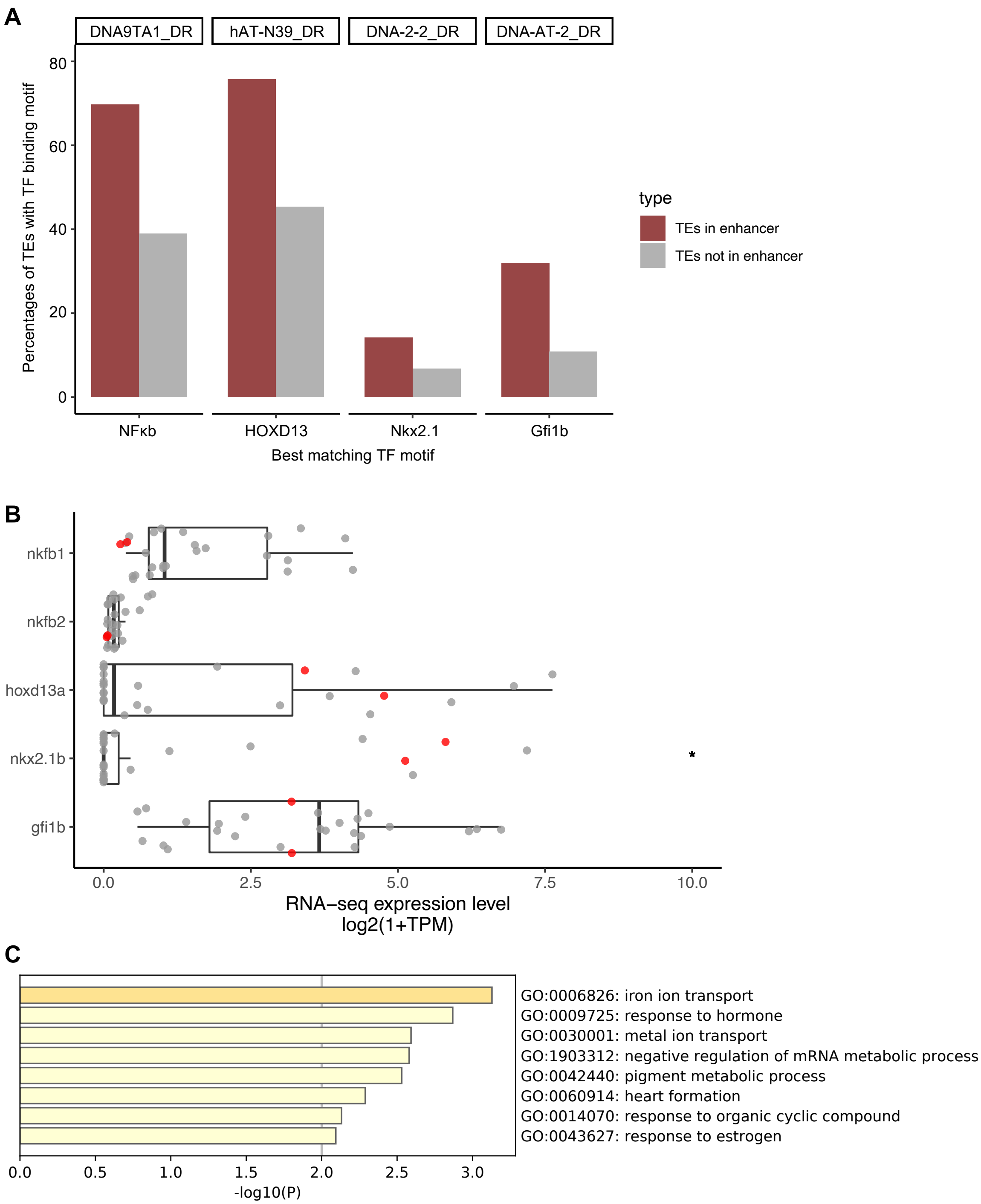

Supplemental Figure S5. Motif analysis of enriched TE subfamilies in testis-specific enhancer regions**.** **(***A*) Percentages of TE fragments containing a binding motif. (*B*) RNA-seq expression level of transcription factor genes. (*C*) Gene Ontology analysis of TE fragments of DNA-2-2_DR located within testis-specific enhancer regions.

Supplemental Figure S6

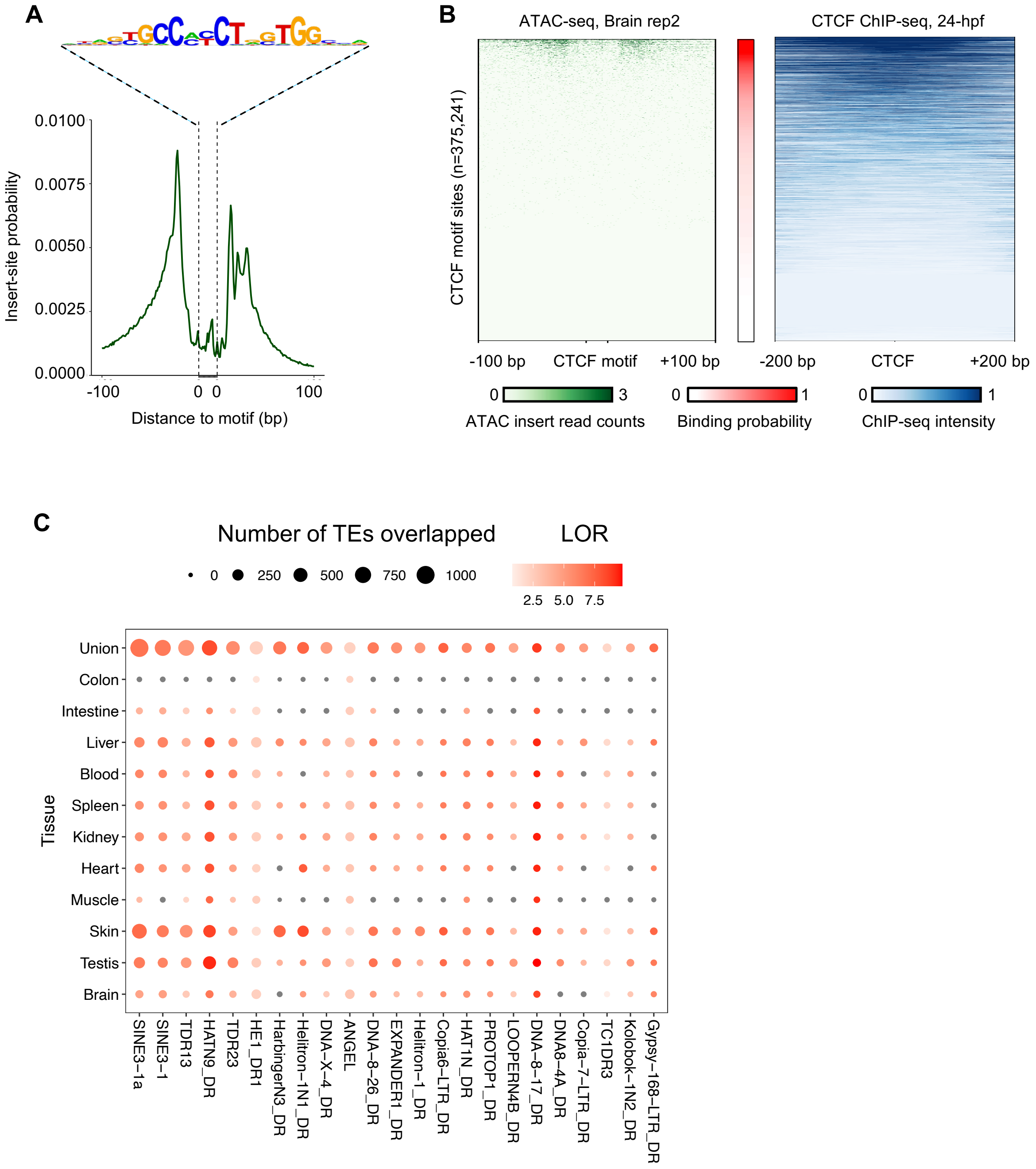

Supplemental Figure 6. TE subfamily enrichment for the CTCF binding sites. (*A*) CTCF footprint analysis. Aggregate ATAC-seq footprint for CTCF (motif shown above) generated over binding sites in the zebrafish genome. (*B*) CTCF predicted binding probability inferred from ATAC-seq data (middle), ATAC-seq insert read counts over CTCF motifs (left), and the CTCF ChIP-seq data for 24 hours post-fertilization embryos (right). (*C*) Heatmap of the TE subfamily enrichment for the CTCF bound sites. The color scale and the size of dots represent LOR and the number of TEs overlapped with CTCF bound sites, respectively.

Supplemental Figure S7

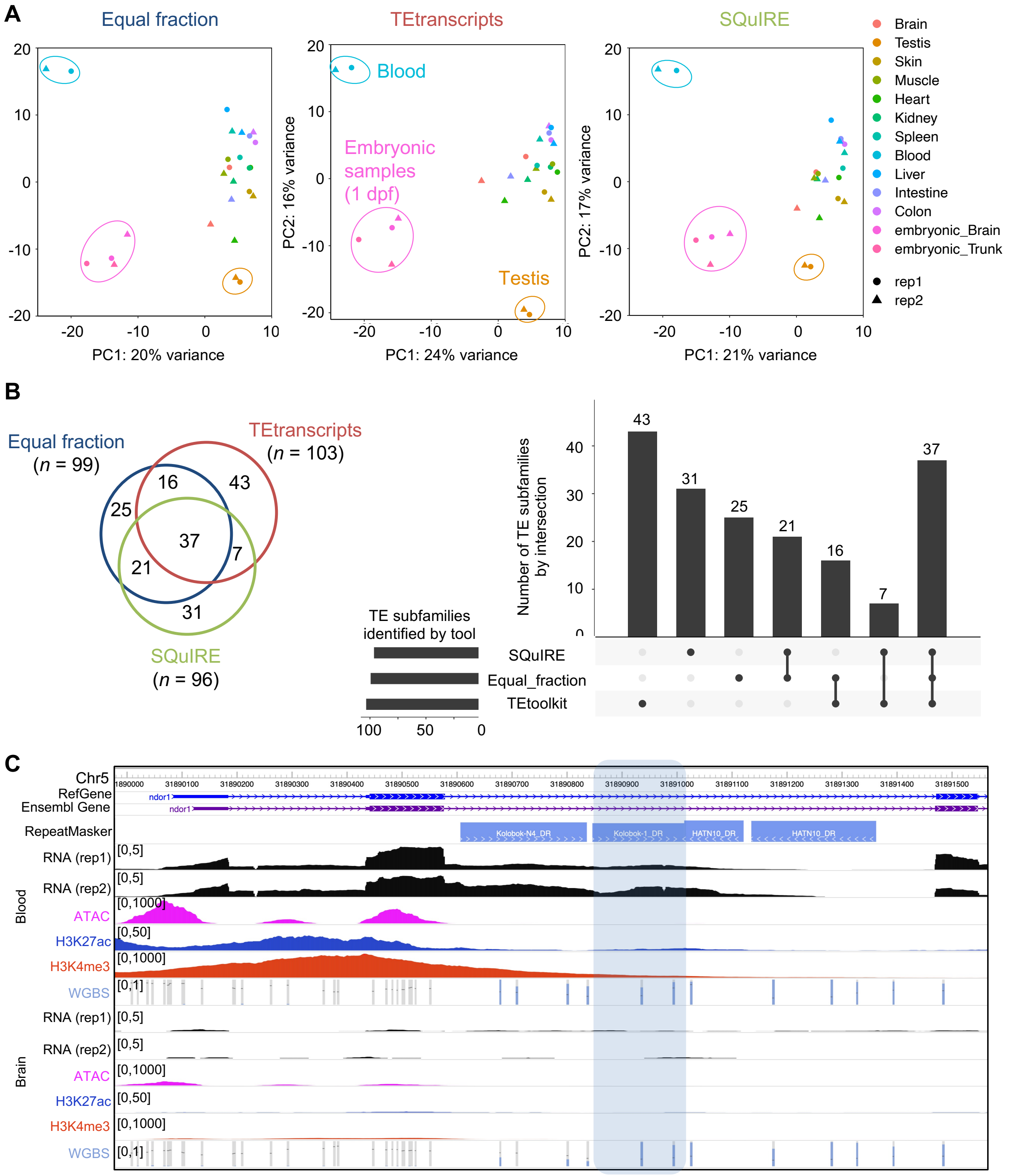

Supplemental Figure 7. TE expression quantification benchmarking. (*A*) PCA plots of the TE expression patterns using the three different methods of assigning multi-mapped reads. The expression levels of all TE subfamilies were used. (*B*) A Venn diagram and UpSet plot of tissue-specific expression of TE subfamilies. (*C*) An example TE locus that shows blood-specific expression. The Kolobok-1_DR subfamily was identified to be expressed blood specifically, but RNA-seq tracks suggest that the reads are due to the intron retention from the *ndor1* gene.

Supplemental Figure S8

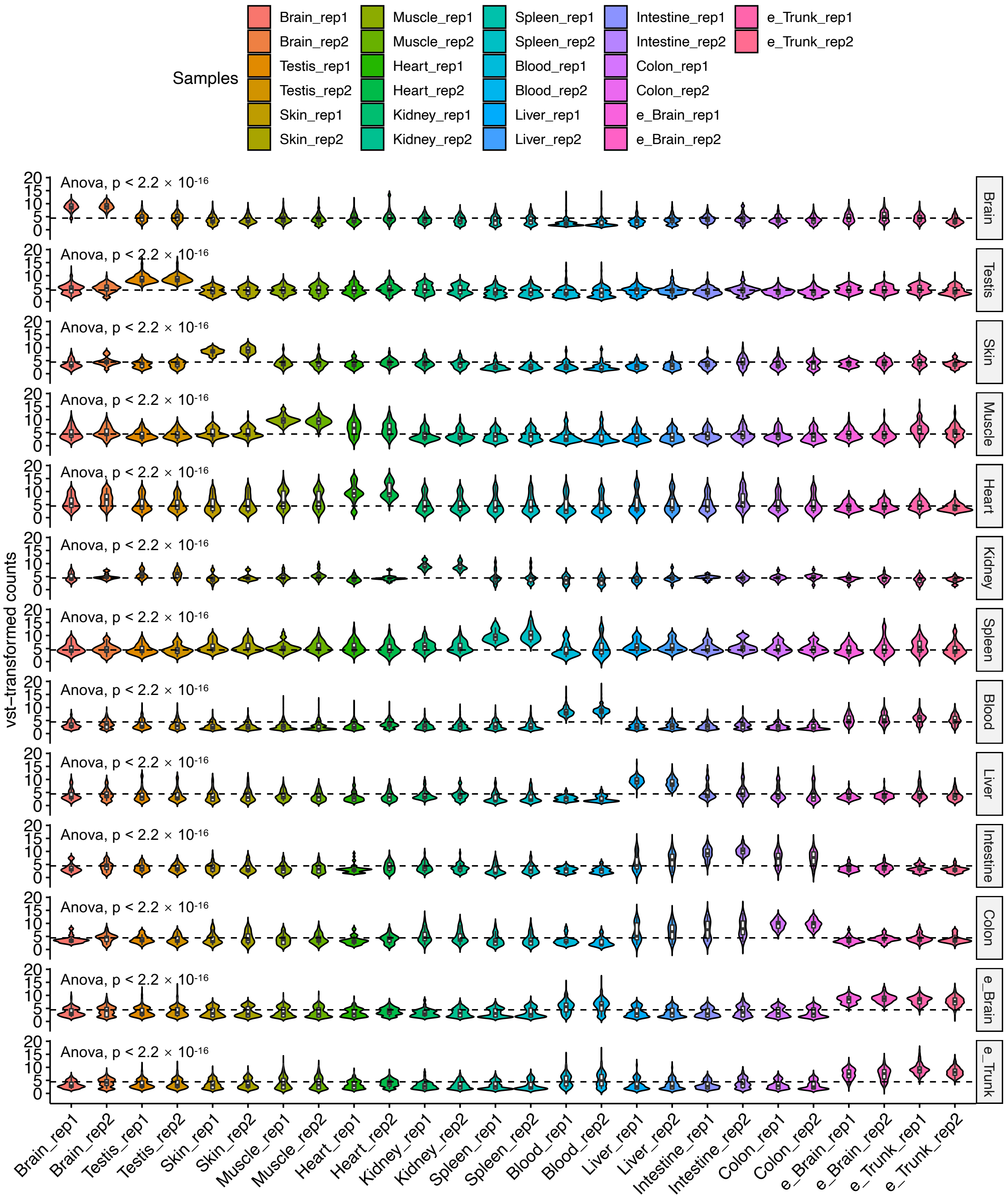

Supplemental Figure 8. Expression level distribution of tissue-specific TE transcripts across all tissues. Violin-box plot showing expression level of TE transcripts in all individual samples grouped by tissue specificity on the rows. Expression level is presented as vst-transformed counts (vst - variance stabilizing transformation). Dashed lines stand for the average value of vst-transformed count of all tissue-specific TE transcripts. Between group mean difference is tested by ANOVA multiple comparisons.

Supplemental Figure S9

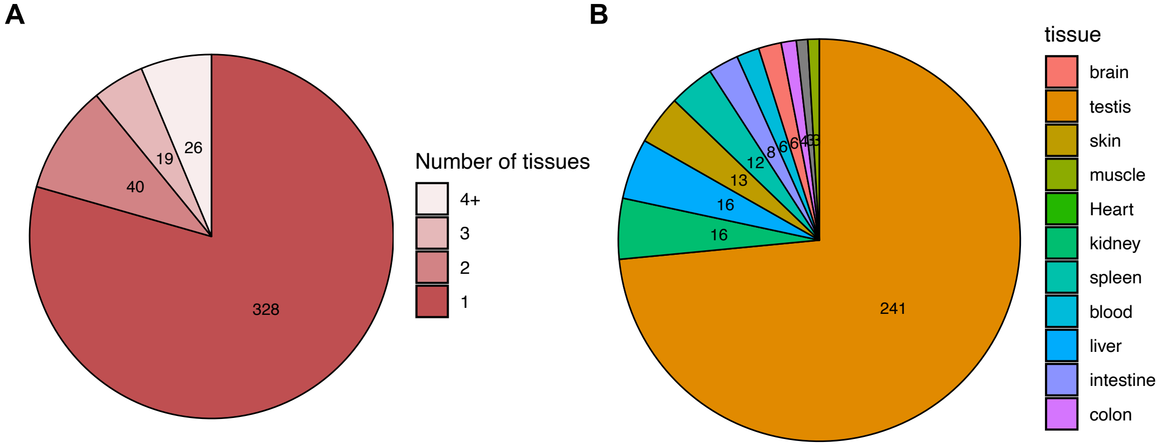

Supplemental Figure 9. Number of tissues expressing TE transcripts. (*A*) Number of expressed tissues per TE-TSSs. (*B*) Number of tissue-specific TE-TSSs.

Supplemental Figure S10

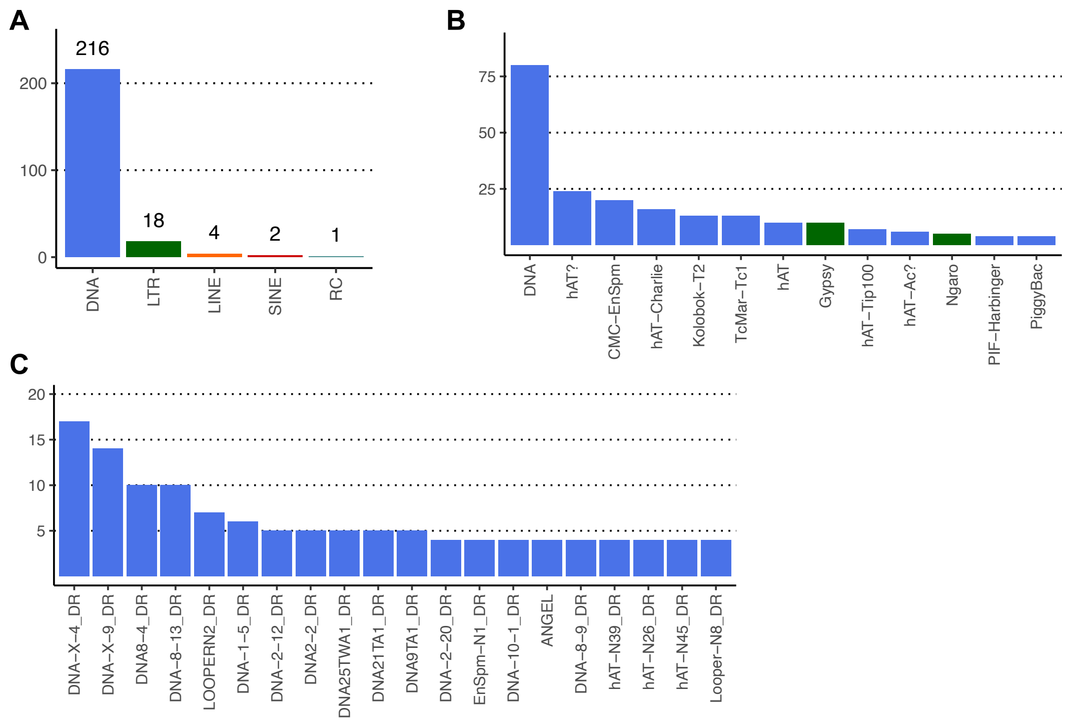

Supplemental Figure 10. Testis-specific alternative promoters derived from TEs. (*A-C*) The number of TE classes (*A*), families (*B*), and subfamilies (*C*) that contributed to testis-specific alternative promoters. Only TE families and subfamilies ≥ 4 instances are shown in *(B)* and (*C*), respectively.

Supplemental Figure S11

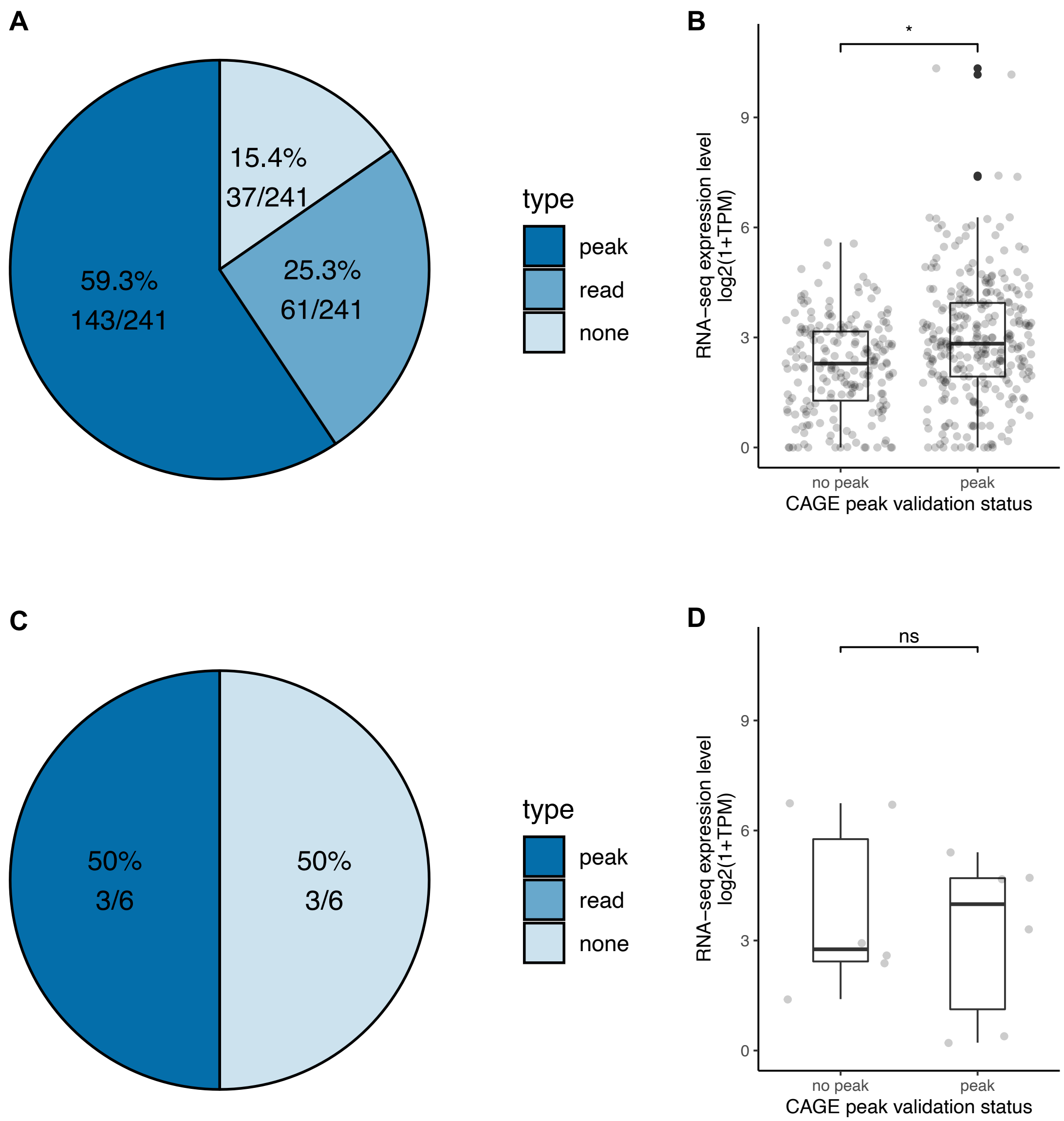

Supplemental Figure 11. nanoCAGE validation of testis-specific and brain-specific TE-TSS. (*A*) Percentages of 241 testis-specific TE-TSSs validated by nanoCAGE reads and peaks. (*B*) RNA-seq expression level of testis-specific TE-TSSs as a function of nanoCAGE peak support (Two-sided Wilcoxon test, *: p-value <0.05, ns: not significant). (*C*) Percentages of 6 brain-specific TE-TSSs validated by nanoCAGE reads and peaks. (*D*) RNA-seq expression level of brain-specific TE-TSSs as a function of nanoCAGE peak support.

Supplemental Figure S12

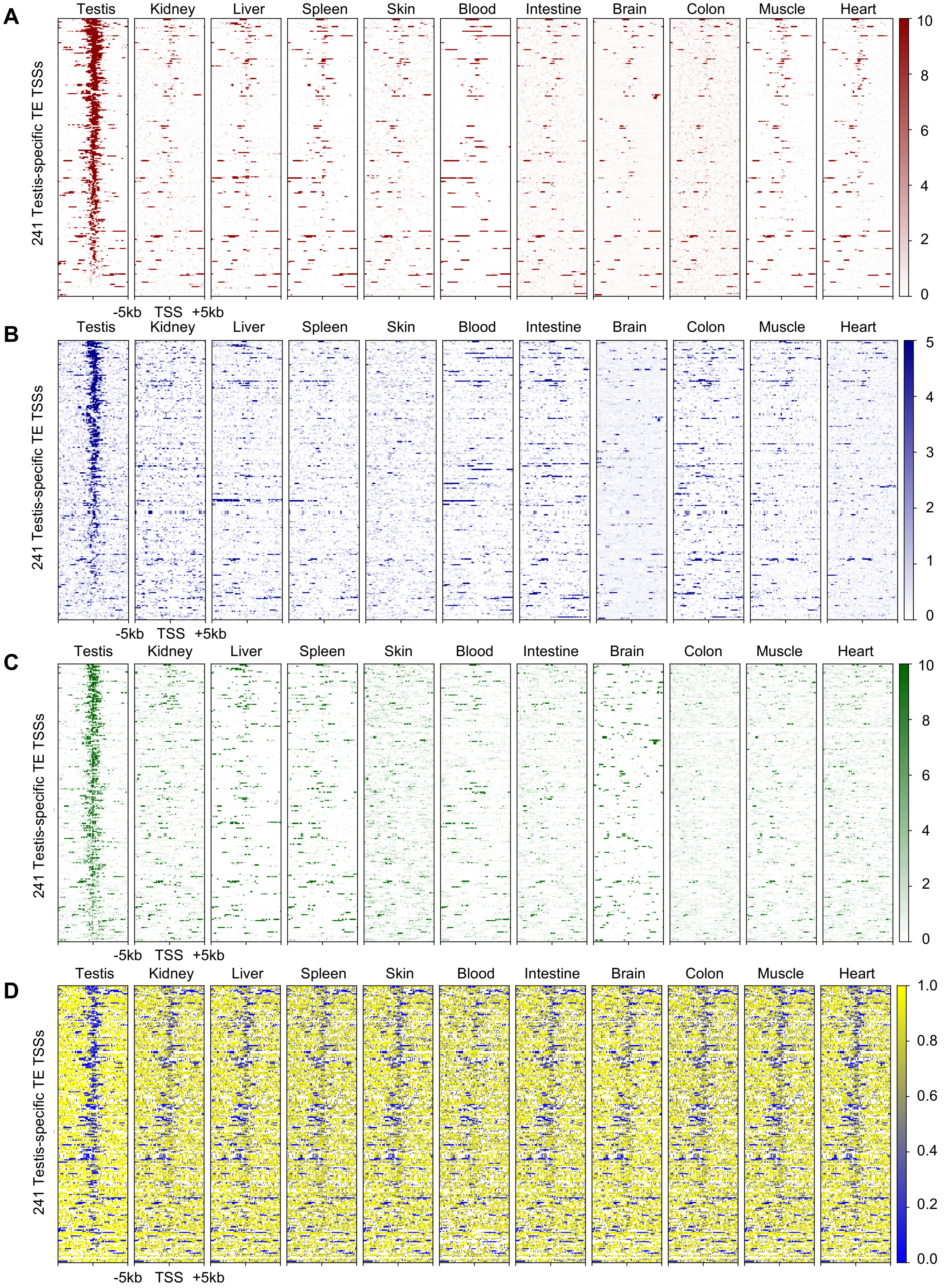

Supplemental Figure 12. Epigenetic landscape of testis-specific TE-TSS across all tissues. (*A-D*) Epigenetic signal at 241 testis-specific TE-TSSs and their flanking 10 kb regions are shown in heatmaps. All four heatmaps are ordered by total H3K4me3 signal at testis-specific TE-TSSs using testis data. (*A*) H3K4me3. (*B*) H3K27ac. (*C*) ATAC. (*D*) WGBS.

Supplemental Figure S13

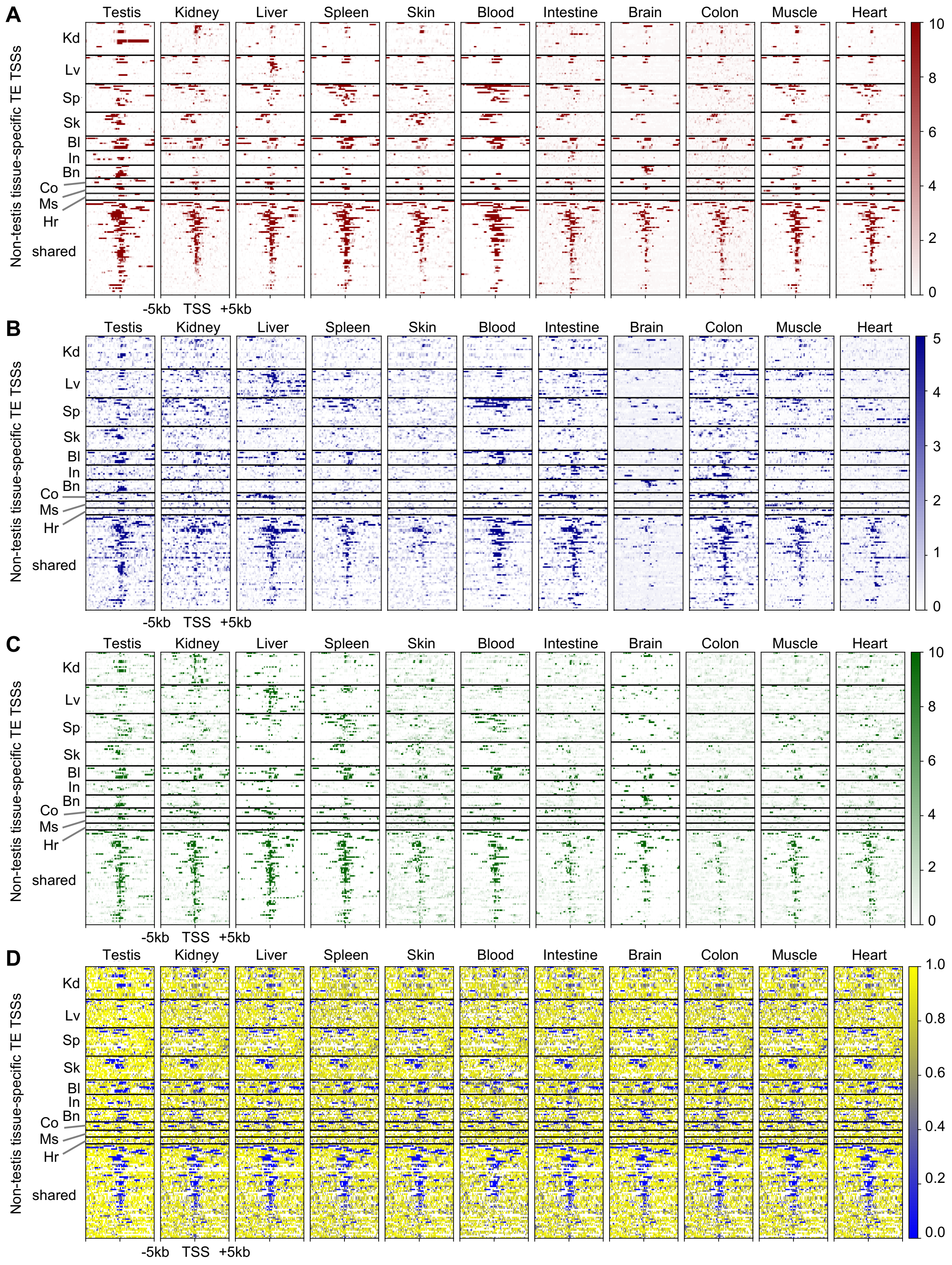

Supplemental Figure 13. Epigenetic landscape of non-testis tissue-specific TE-TSS across all tissues. (*A-D*) Epigenetic signal of 172 TE-TSSs and their flanking 10 kb regions are shown in heatmaps (111 tissue-specific TE-TSSs, 61 shared TE-TSSs). Tissue-specific TE-TSSs are defined using 10 adult tissues excluding testis. Heatmaps at the tissue-specific TE-TSSs are ordered by total H3K4me3 signal using the corresponding tissue data. Heatmaps at the shared TE-TSSs are ordered by total H3K4me3 signal using kidney data. (*A*) H3K4me3. (*B*) H3K27ac. (*C*) ATAC. (*D*) WGBS.

Supplemental Figure S14

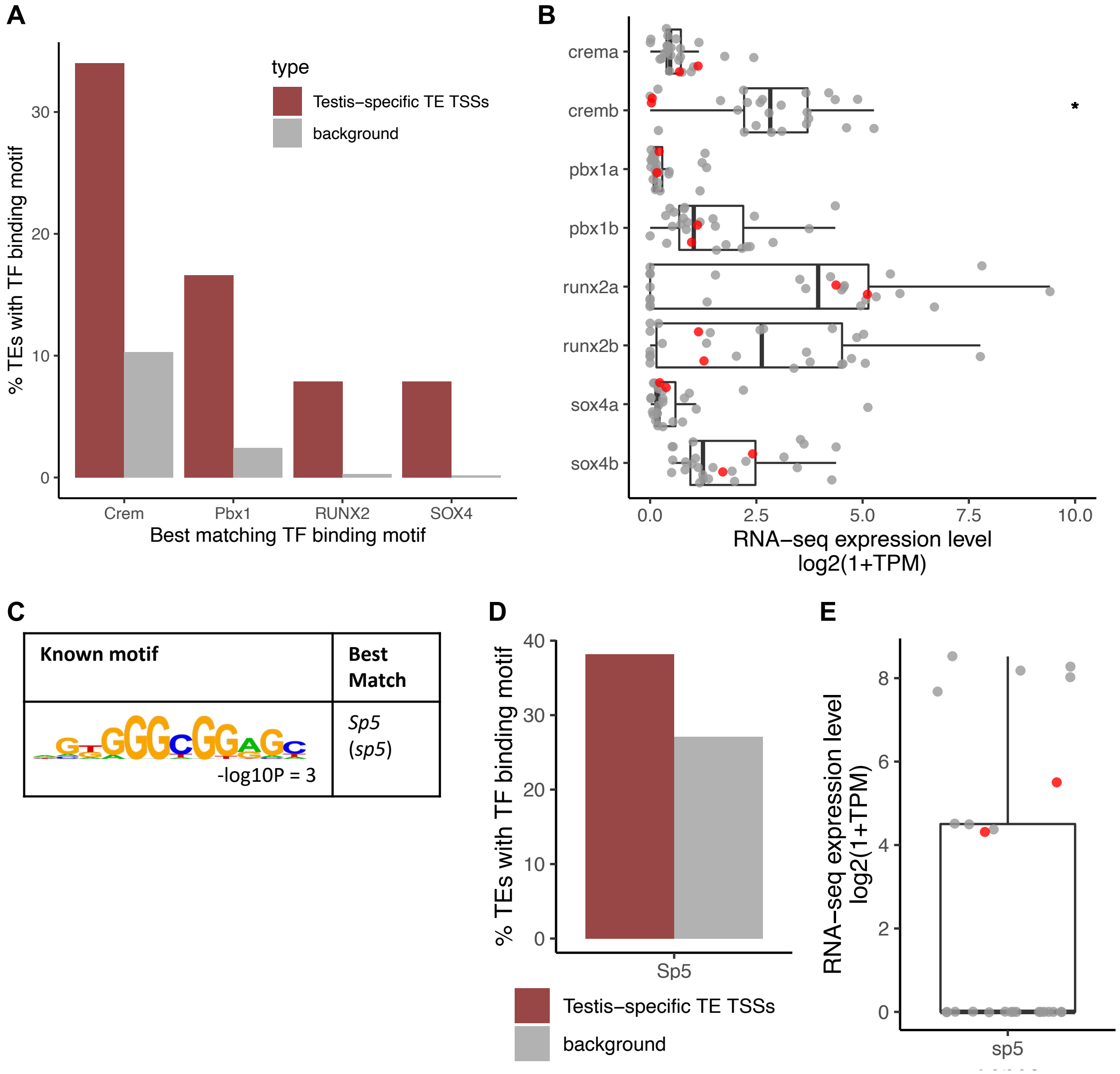

Supplemental Figure 14. Motif analysis of testis-specific TE-TSSs. (*A*) Percentages of testis-specific TE-TSSs containing a *de novo* binding motif. (*B*) RNA-seq expression level of transcription factor genes of *de novo* motif. (*C*) Known motif of testis-specific TE-TSSs. (*D*) Percentages of testis-specific TE-TSSs containing a known binding motif. (*E*) RNA-seq expression level of *sp5* transcription factor gene of the known motif across samples.

Supplemental Figure S15

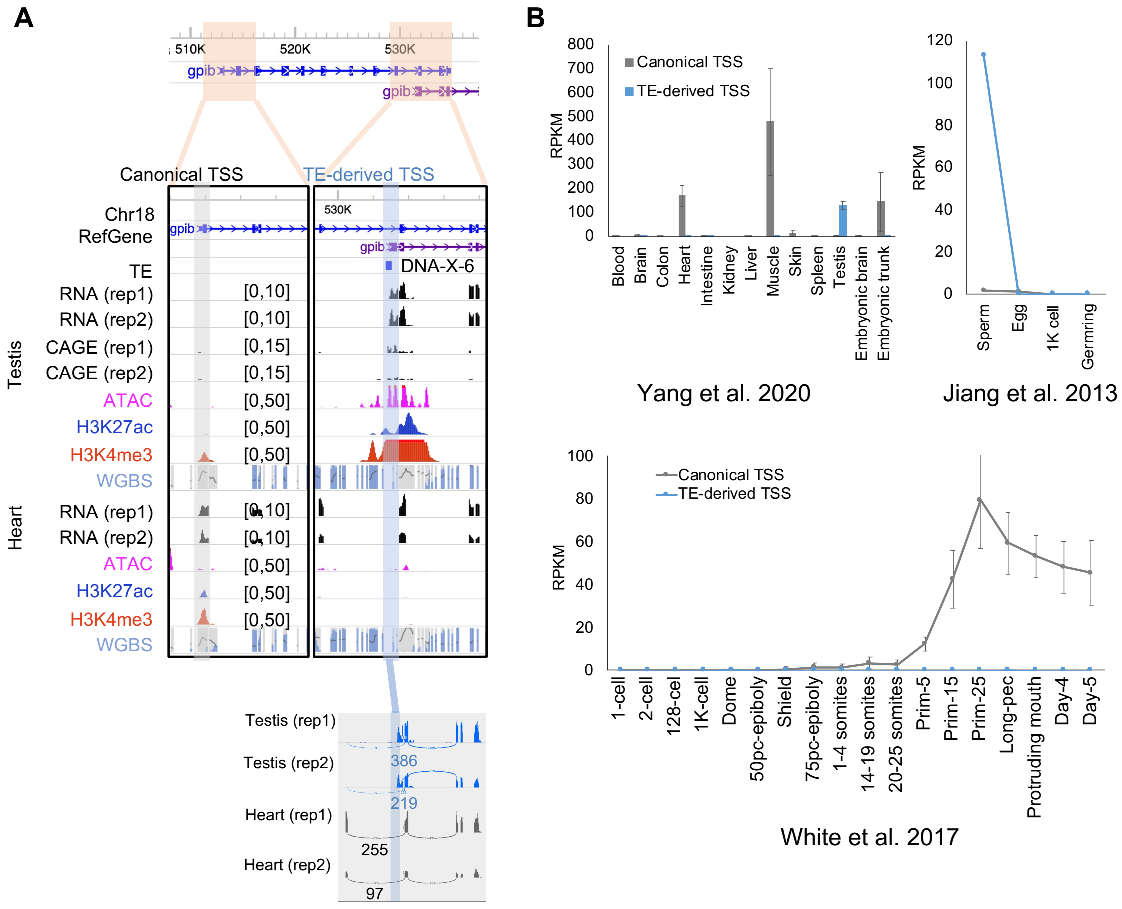

Supplemental Figure 15. Testis-specific TE-TSS transcript of *gpib*. (*A*) An Epigenome Browser view of TE-derived alternative promoter and canonical promoter of the *gpib* gene. A sashimi plot showing the numbers of RNA-seq reads spanning exon-exon junctions is below. (*B*) Plots of canonical and TE-derived TSS usages for the *gpib* gene in the different zebrafish tissues and developmental stages.

Supplemental Figure S16

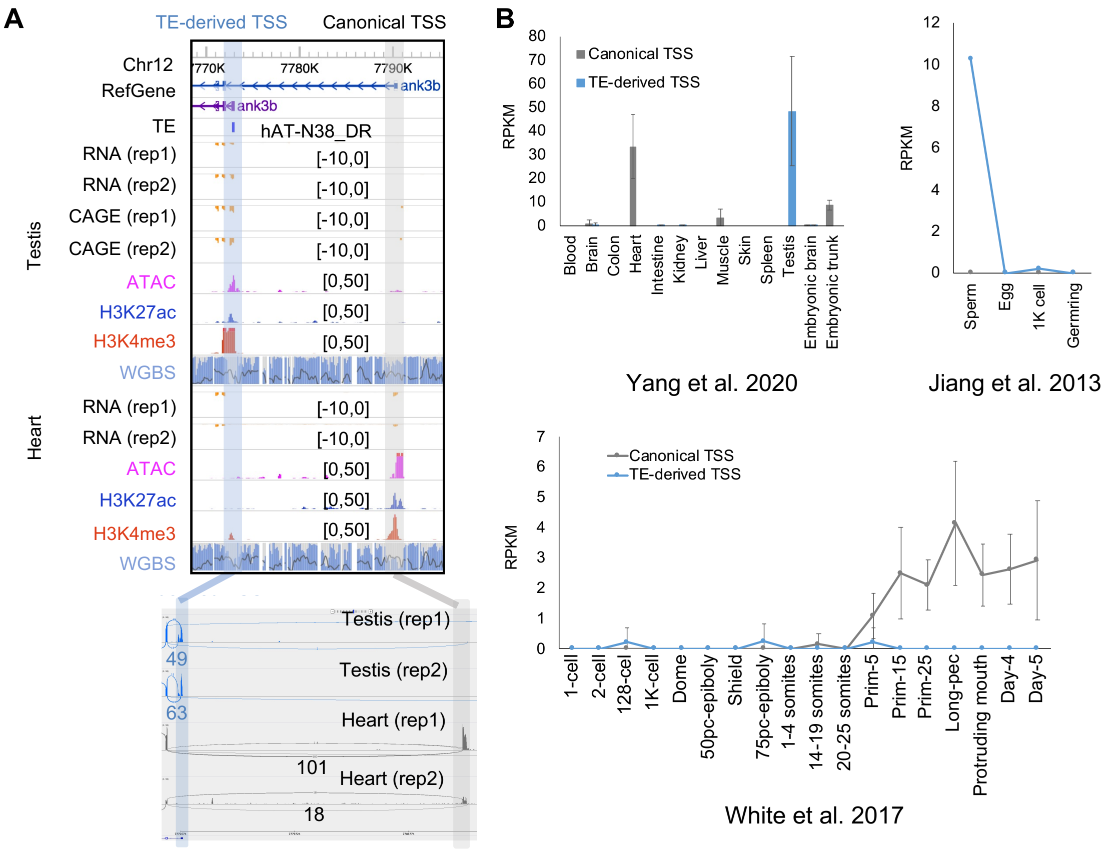

Supplemental Figure 16. Testis-specific TE-TSS transcripts of *ank3b*. (*A*) Epigenome Browser views of TE-derived alternative promoter and canonical promoter of *ank3b*, with Sashimi plot showing the numbers of RNA-seq reads spanning exon-exon junctions. (*B*) Plots of canonical and TE-derived TSS usages for *ank3b* in the different zebrafish tissues and developmental stages.

Supplemental Figure S17

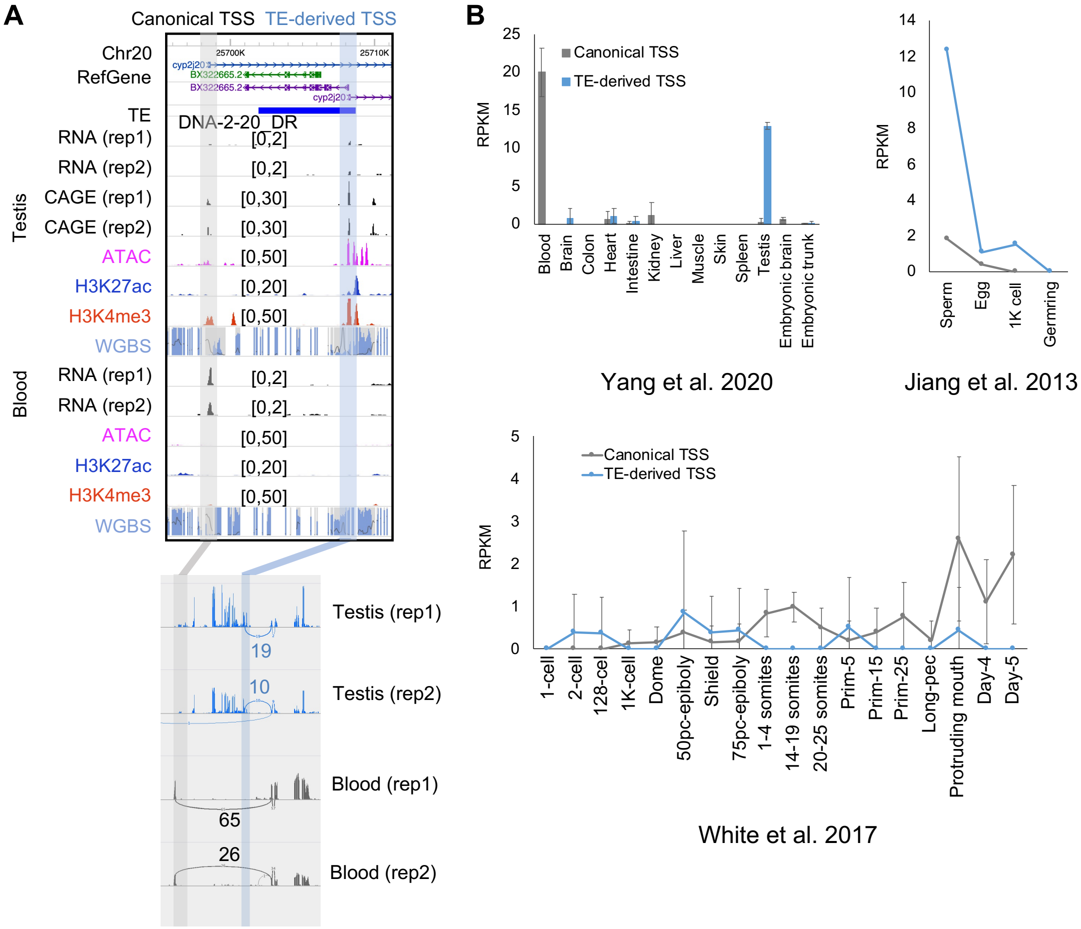

Supplemental Figure 17. Testis-specific TE-TSS transcripts of *cyp2j20*. (*A*) Epigenome Browser views of TE-derived alternative promoter and canonical promoter of *cyp2j20*, with Sashimi plot showing the numbers of RNA-seq reads spanning exon-exon junctions. (*B*) Plots of canonical and TE-derived TSS usages for *cyp2j20* in the different zebrafish tissues and developmental stages.

Supplemental Figure S18

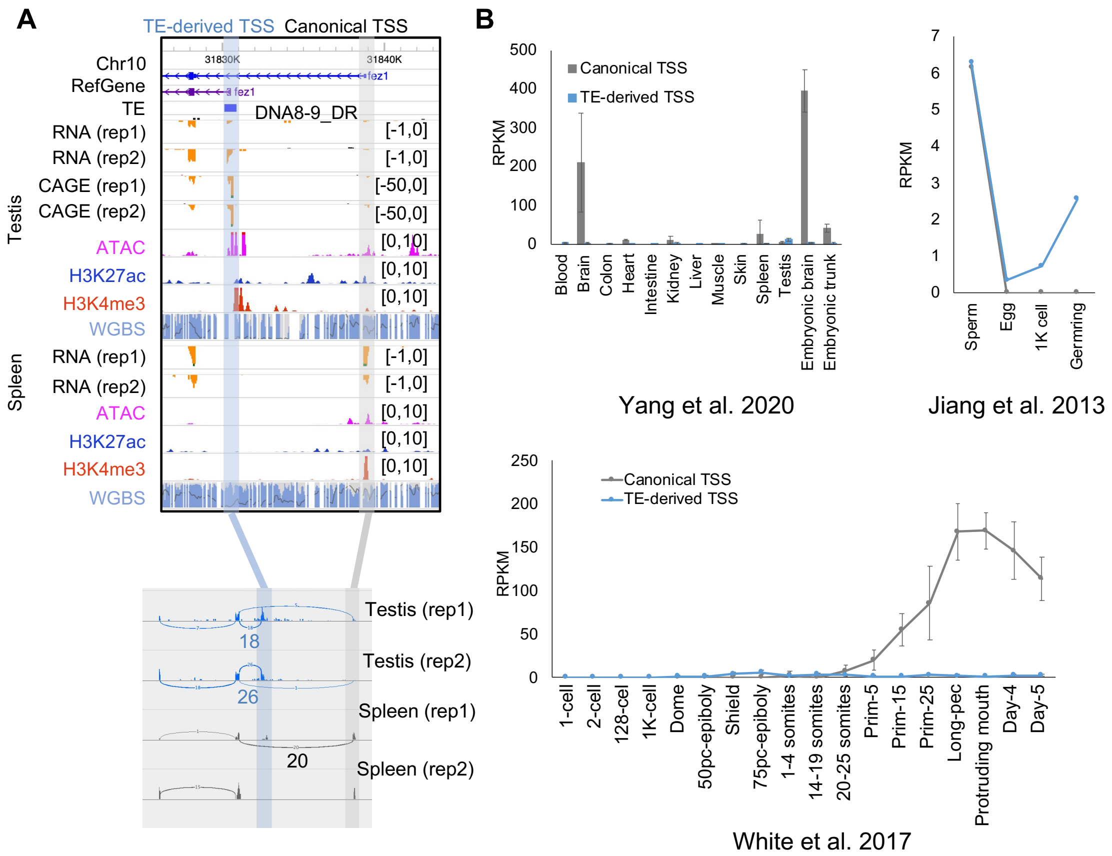

Supplemental Figure 18. Testis-specific TE-TSS transcripts of *fez1*. (*A*) Epigenome Browser views of TE-derived alternative promoter and canonical promoter of *fez1*, with Sashimi plot showing the numbers of RNA-seq reads spanning exon-exon junctions. (*B*) Plots of canonical and TE-derived TSS usages for *fez1* in the different zebrafish tissues and developmental stages.

Supplemental Figure S19

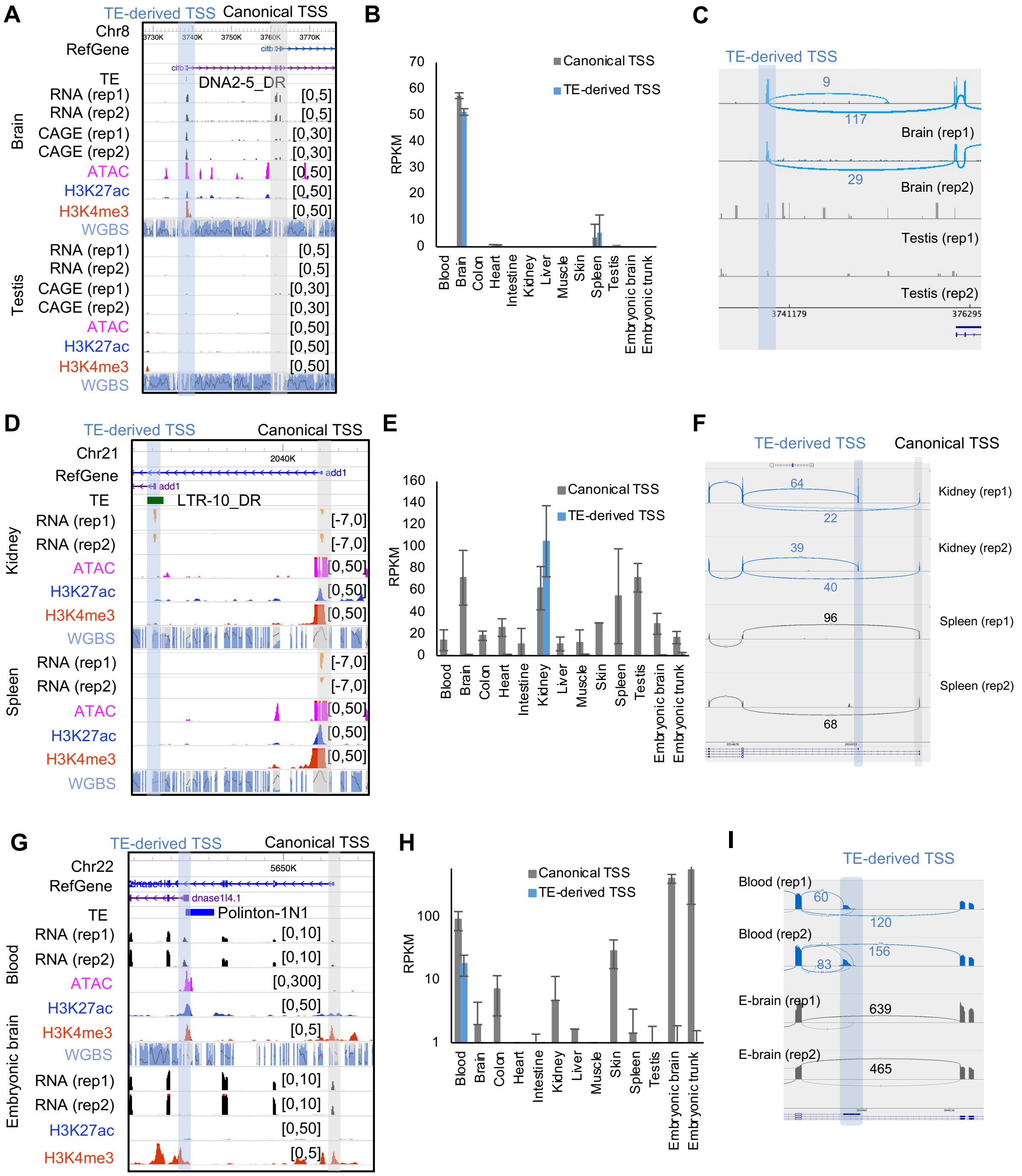

Supplemental Figure 19. Tissue-specific TE-TSSs transcripts in non-testis samples. (*A,D,G*) Epigenome Browser views of TE-derived alternative promoter and canonical promoter of the genes *citb* (*A*), *add1* (*D*), and *dnase1l4.1* (*G*). (*B,E,H*) Plots of canonical and TE-derived TSS usages for the genes *citb* (*B*), *add1* (*E*), and *dnase1l4.1* (*H*) in the different zebrafish tissues and developmental stages. (*C,F,I*) Sashimi plots showing the numbers of RNA-seq reads spanning exon-exon junctions of the genes *citb* (*C*), *add1* (*F*), and *dnase1l4.1* (*I*).

Supplemental Figure S20

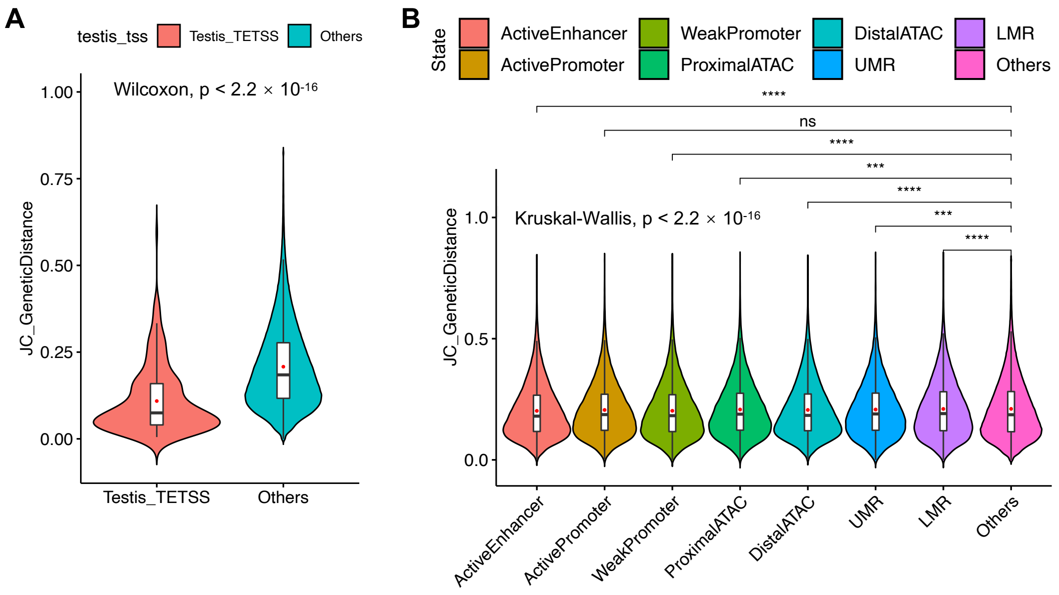

Supplemental Figure 20. Age distribution of TEs contributing to TE-TSS and CREs. (*A*) Distribution of Jukes-Cantor distance of TEs overlapping with active regions including active enhancer, active promoter, weak promoter, proximal ATAC-seq signal, distal ATAC-seq signal, UMR, and LMR. TEs overlapping with the "Others" category is not labeled as any active state in any tissues available in our dataset. Middle lines inside boxes show the median, while red dots show the mean of the age distributions. ns: not significant as $p > 0.05$, ***: $p \leq 0.001$, ****: $p \leq 0.0001$. (*B*) Distribution of Jukes-Cantor distance of TEs whether contributing to testis TE-TSS or not.
